# Rat intersubjective decisions are encoded by frequency‐specific oscillatory contexts

**DOI:** 10.1002/brb3.710

**Published:** 2017-05-05

**Authors:** Jana Schaich Borg, Sanvesh Srivastava, Lizhen Lin, Joseph Heffner, David Dunson, Kafui Dzirasa, Luis de Lecea

**Affiliations:** ^1^ Social Science Research InstituteDuke UniversityDurhamNCUSA; ^2^ Duke Institute for Brain SciencesDuke UniversityDurhamNCUSA; ^3^ Department of Psychiatry and Behavioral SciencesStanford UniversityStanfordCAUSA; ^4^ Department of Statistics and Actuarial ScienceUniversity of IowaIowa CityIAUSA; ^5^ Department of Applied and Computational Mathematics and StatisticsUniversity of Notre DameNotre DameINUSA; ^6^ Department of Psychology, Cognitive Linguistic and Psychological SciencesBrown UniversityProvidenceRIUSA; ^7^ Department of Statistical ScienceDuke UniversityDurhamNCUSA; ^8^ Department of Psychiatry and Behavioral SciencesDuke University Medical CenterDurhamNCUSA; ^9^ Department of NeurobiologyDuke University Medical CenterDurhamNCUSA; ^10^ Department of NeurosurgeryDuke University Medical CenterDurhamNCUSA; ^11^ Department of Biomedical EngineeringDuke UniversityDurhamNCUSA

**Keywords:** empathy, local field potential, oscillations, social behavior

## Abstract

**Introduction:**

It is unknown how the brain coordinates decisions to withstand personal costs in order to prevent other individuals’ distress. Here we test whether local field potential (LFP) oscillations between brain regions create “neural contexts” that select specific brain functions and encode the outcomes of these types of intersubjective decisions.

**Methods:**

Rats participated in an “Intersubjective Avoidance Test” (IAT) that tested rats’ willingness to enter an innately aversive chamber to prevent another rat from getting shocked. c‐Fos immunoreactivity was used to screen for brain regions involved in IAT performance. Multi‐site local field potential (LFP) recordings were collected simultaneously and bilaterally from five brain regions implicated in the c‐Fos studies while rats made decisions in the IAT. Local field potential recordings were analyzed using an elastic net penalized regression framework.

**Results:**

Rats voluntarily entered an innately aversive chamber to prevent another rat from getting shocked, and c‐Fos immunoreactivity in brain regions known to be involved in human empathy—including the anterior cingulate, insula, orbital frontal cortex, and amygdala—correlated with the magnitude of “intersubjective avoidance” each rat displayed. Local field potential recordings revealed that optimal accounts of rats’ performance in the task require specific frequencies of LFP oscillations between brain regions *in addition to* specific frequencies of LFP oscillations within brain regions. Alpha and low gamma coherence between spatially distributed brain regions predicts more intersubjective avoidance, while theta and high gamma coherence between a separate subset of brain regions predicts less intersubjective avoidance. Phase relationship analyses indicated that choice‐relevant coherence in the alpha range reflects information passed from the amygdala to cortical structures, while coherence in the theta range reflects information passed in the reverse direction.

**Conclusion:**

These results indicate that the frequency‐specific “neural context” surrounding brain regions involved in social cognition encodes outcomes of decisions that affect others, above and beyond signals from any set of brain regions in isolation.

## 1 Introduction

“Empathy” is an innate, fundamental phenomenon that confers powerful evolutionary advantage. Empathy‐motivated relationships increase individuals’ reproductive success (Seyfarth & Cheney, 2012) and decrease mortality (Holt‐Lunstad, Smith, & Layton, 2010), which might be related to observations that empathy inhibits aggression, motivates cooperation, and facilitates moral principles (De Waal, 2008; Eisenberg & Morris, 2001). Decisions and actions based on empathy are posited to be responsible for the evolution of humans’ advanced cognitive abilities (Burkart et al., 2014; De Waal, 2008). Understanding how empathy influences decisions and actions will provide an essential window into the evolutionary trajectories that make us uniquely human, and provide insight into how to enhance prosocial behavior and decrease human violence.

The subjective experience of making an empathic decision often gives a decider the sense that the decision is the result of one continuous cognitive operation. However, this subjective experience may be a misleading guide of what is actually happening in the brain when an empathic decision is made. Decision‐making is the result of multiple information processing systems acting in parallel (Barrett & Satpute, 2013; Doya, 2008). Neither decision‐making nor social processing is localized to a single brain region (Barrett & Satpute, 2013; Ruff & Fehr, 2014; Stanley & Adolphs, 2013; Teles, Almeida, Lopes, & Oliveira, 2015). Furthermore, most individual brain regions involved in decision‐making and social processing are responsible for several different cognitive subfunctions (Betti & Aglioti, 2016). Mechanisms must exist to dynamically select the specific function, or functions, a given brain region plays in social cognition, especially if the same brain region plays multiple functions simultaneously. It has been posited that the function a brain region executes in social situations might be based on its “neural context”, or its interaction with the activity of brain regions it is connected to at that time (Goodson & Kabelik, 2009; McIntosh, 1999; Park & Friston, 2013; Pessoa, 2014). If true, empathic decisions may be reflected by the overall coordination of activity across different brain regions rather than, or in addition to, the activity of a single social processing brain region (Goodson & Kabelik, 2009).

Neural oscillations provide a window into coordinated neural activity that could create functionally‐specific neural context. Information passed between a pair of brain regions can be preferentially amplified or communicated by coordinating the oscillatory amplitudes and phases of transmembrane currents in groups of local neurons within those brain regions (Akam & Kullmann, 2014; Buschman & Kastner, 2015; Henry, Herrmann, & Obleser, 2014; Voytek & Knight, 2015). Such oscillations are observable through local field potentials (LFPs; Başar, Başar‐Eroglu, Karakaş, & Schürmann, 2001), which are extracellular brain potentials comprised of weighted spatial averages of large local populations of neurons’ transmembrane currents. LFPs are comprised of oscillations of different frequency bands ranging in width (ie: “theta band” oscillations are typically 5 Hz in width and span 4–8 Hz; Lewis, 2012) that are believed to have different functions (Akam & Kullmann, 2014). Local field potential “power” is a measure of the magnitude of these oscillations at a single location. Local field potential “coherence” is a measure of temporally synchronized versions of these oscillations between more than one brain location (Canolty et al., 2010; Wang, 2010). Given the role coordinated oscillations have been shown to play in information transfer (Akam & Kullmann, 2014; Buschman & Kastner, 2015; Henry et al., 2014; Voytek & Knight, 2015), an efficient mechanism for coordinating neural context would be to orchestrate selective frequencies of LFP coherence between brain regions. Thus, we hypothesized in this study that individual judgments to avoid another individual's pain would be partially encoded by selective frequencies of coherence between social cognition brain regions.

It is difficult to test relationships between coherence and intersubjective decision‐making in humans due to the ethical and methodological challenges of recording LFPs from multiple spatially distributed regions in the human brain. It is similarly difficult to test relationships between coherence and intersubjective decision‐making in rodents, due to the challenges in developing appropriate behavioral, recording, and analysis techniques for assessing LFP activity in multiple brain regions simultaneously while rodents make intersubjective decisions. To manage those challenges, we begin in this study by developing a test of rat intersubjective decision‐making that models empathic decision‐making in humans. A common method of studying animals’ cost‐benefit decisions between competing alternatives is to examine animals’ locomotor choices to avoid or approach salient stimuli (Hirayama, Moroz, Hatcher, & Gillette, 2014). When an animal avoids a stimulus, that stimulus is interpreted as causing an aversive experience for the decision‐maker (Corsini, 1999). Building on this method, we designed the “Intersubjective Avoidance” (IA) test to measure how much an Observing rat will avoid locations paired with another rat's distress. “Intersubjective”, in this case, refers to the fact that negatively‐valenced affect has to be transferred between the rat that receives pain and the Observing rat in order for the Observer to be motivated to exhibit avoidance.

Taking advantage of the IA test, we used c‐Fos mapping to identify brain regions that encode the extent to which one rat avoided other rats’ pain. We then designed a surgical strategy to record LFPs from all of these areas simultaneously while rats were making intersubjective decisions, analyzed power oscillations within each region and coherence oscillations between each pair of regions, and applied a machine‐learning framework to determine what frequencies of LFP power and coherence encoded the outcomes of those decisions.

Supporting our hypothesis, we found that the optimal description of rats’ decisions about how to respond to another rat's pain required measurements of local oscillations within—*and* measurements of long‐distance oscillations *between*—the anterior cingulate, anterior insula, orbitofrontal cortex, basolateral amygdala, and olfactory amygdala. Furthermore, intersubjective decision‐encoding oscillations were observed primarily when rats were witnessing another rat get shocked, rather than when rats had already chosen to avoid another rat's pain, supporting the inference that the oscillations were related to the integration of social cues with neural decision‐making machinery, rather than some type of generalized arousal. These results suggest that the brain networks involved in rat intersubjective decision‐making may be evolutionarily conserved, and provide evidence that intersubjective decisions are encoded through interactions between brain regions as well as through isolated neural activity.

## 2 Materials and Methods

### 2.1 Subjects

Male Wistar rats from Charles River (approximately 150 g upon delivery) were pair‐housed in one of three animal facilities (two at Stanford, one at Duke), and maintained on a reverse 12‐hr light‐dark cycle with free access to food and water. Animals were handled for a minimum of 5 days before testing. Animals were 8–20 weeks old when the experiments commenced (cohorts 1–4 were approximately 9, 20, 10, 14 weeks old, respectively; the arousal cohort was approximately 8 weeks old; the electrophysiology cohort was approximately 8–9 weeks old when the surgeries were implemented and 10 weeks old when behavioral habituation commenced). All animal procedures were approved by the Stanford University or Duke University Institutional Animal Care and Use Committees and were in accordance with the NIH guidelines for the Care and Use of Laboratory Animals.

### 2.2 Procedure

Experiments were conducted during the animals’ circadian dark cycle. After testing, animals were transferred from the testing room to a separate holding room until all of the animals for the day had been run. All testing apparatuses were cleaned with Nature's Miracle^®^ between sessions.

### 2.3 Intersubjective avoidance apparatus

**Figure 1:**
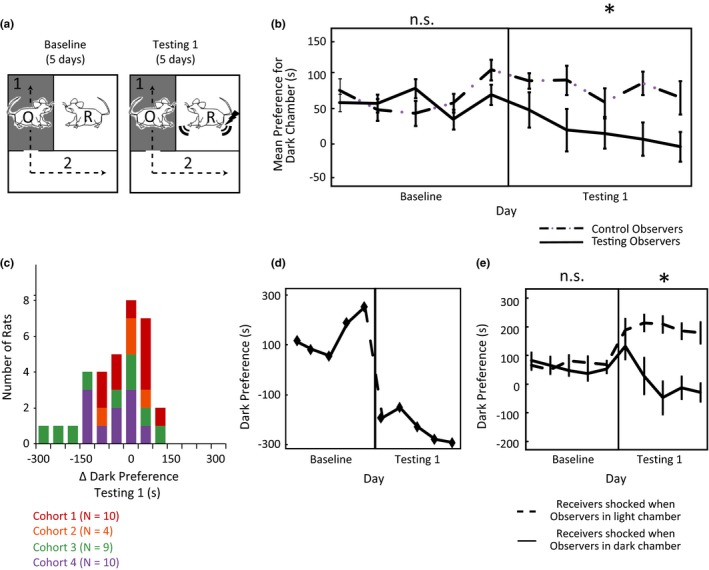
Receiver distress is aversive and motivates avoidance. (a) Intersubjective Avoidance (IA) test design: Baseline and Testing 1. (b) Testing Observers (*N *= 33) reduce their light avoidance/dark preference during Testing 1. *Testing v. Control Observers, *p *<* *.01 in repeated measures ANOVA (with cohort as a covariate). (c) Histogram of all Observers’ behavior during Testing 1 (color‐coded by cohort). Intersubjective avoidance was demonstrated whenever an Observer's dark preference was reduced during testing (Δ Dark Preference is negative). (d) IA of “Natural Avoider” shown in Movie S1. (e) Observers’ IA is spatially specific; Observers’ dark preference increased when going into the light chamber was paired with shocks to Receivers (*N *= 6), but decreased when going into the dark chamber was paired with shocks to Receivers (*N *= 6). * Experimental group × testing phase interaction in repeated measures ANOVA,* p *<* *.05. All error bars indicate s.e.m.

The testing apparatus, adapted from a previous report (Preobrazhenskaya & Simonov, 1970), was made of clear acrylic plastic. One inner chamber was 12.0″ L × 9.5″ W to fit a metal grid floor purchased from Med Associates (ENV‐008). Two outer chambers (12.0″ L × 7.0″ W and 17.0″  × 7″) each shared a separate, but adjacent transparent wall with the inner chamber (Figure 1a). The transparent walls between chambers had holes to allow visual, auditory, olfactory, and nose‐to‐nose tactile contact between the inner chamber and both outer chambers. The outer chambers were connected by an open door (3.0″ W × 4.0″ H). One outer chamber (outer chamber 1 in Figure 1a) had three darkened walls and a black floor made out of LEGOs^®^ for texture, while the other outer chamber (outer chamber 2 in Figure 1a) had two white walls, one transparent wall, and a white plastic floor. One 60W light bulb was placed outside the transparent wall of outer chamber 2 to illuminate it with approximately 1050 lux (Stanford animal facility 1), 950 lux (Stanford animal facility 2) or 350 lux (Duke animal facility); illumination was adjusted so that pilot cohorts preferred the dark chamber by 60–120 s during baseline. In all cases the dark outer chamber was kept at approximately 12 lux. The Receiver's chamber was held at approximately 250 lux.

### 2.4 Intersubjective avoidance (“IA”) test

Each day, the “Receiver” was placed in the single inner chamber, the “Observer” was placed in the white outer chamber, and then the experimenter began recording an overhead video (Figure 1a). The Observer was allowed to pass freely between the two outer chambers for five minutes (starting when the video session was initiated). The test was comprised of three 5‐day phases (and one 1‐day interim phase), and the amount of time the Observer spent in each outer chamber was recorded each day. A given Observer would be paired with a different noncagemate Receiver each day so that no Receiver‐Observer pair was repeated more than once during testing phases of the experiment.

**Figure 2:**
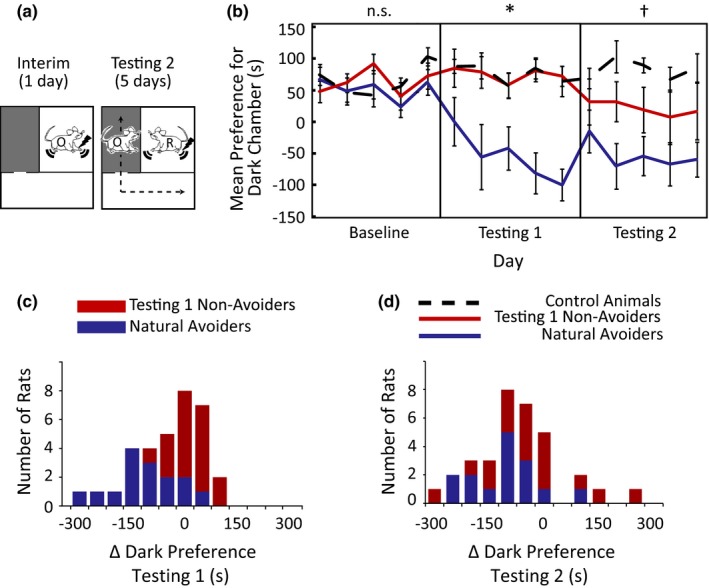
Experience with electrical shock increases Observers’ Intersubjective Avoidance (IA). (a) IA test design: Interim phase and Testing 2. (b) Testing 1 Non‐avoiders reduce their light avoidance and Natural Avoiders maintain their intersubjective avoidance during Testing 2. *Natural Avoiders versus Controls *p *<* *.01; Testing 1 Non‐avoiders versus controls *p *=* *.17. †Natural Avoiders versus Controls *p *<* *.01, Testing 1 Non‐avoiders versus controls, *p *=* *.04. (c) Testing 1 Non‐avoiders and Natural Avoider designations overlaid on Testing 1 behavioral histogram (see 2 for more details). (d) Histogram of all Observers’ behavior during Testing 2. Intersubjective avoidance was demonstrated whenever an Observer's dark preference was reduced during testing (Δ Dark Preference is negative). All error bars indicate s.e.m

After Observers’ were habituated to the apparatus (Deacon, 2006; Whishaw and Kolb, 2005; see Supplementary Methods in Supporting Information for details), the 5‐day Baseline Phase began. During Baseline, Receivers and Observers were allowed to explore their respective environments without disruption. The day after Baseline finished, the 5‐day Testing 1 phase began. During Testing 1, every time an Observer passed into the dark chamber the Receiver would receive 3 quick successive shocks (1.5 mA; 500 ms on/500 ms off/1000 ms on/1000 ms off/500 ms on/500 ms off) every 10 s until the Observer left the dark chamber. Visual inspection of all experimental videos confirmed that Receivers were generally not able to avoid the electrical shocks, although some animals did attempt to do so by holding onto cracks or holes in the ceiling of the Receivers’ chamber. Observers who did not show strong avoidance during Testing Phase 1 were defined as “Testing 1 Non‐avoiders”; all other Observers were defined as “Natural Avoiders”. The day after Testing 1 completed comprised the Interim Phase. During Interim (Figure 2a), “Testing 1 Non‐avoiders” were placed in the inner receiving chamber with no other animal present and given 3 quick successive shocks (1.5 mA; 500 ms on/500 ms off/1000 ms on/1000 ms off/500 ms on/500 ms off) every 30 s for 5 min. “Natural Avoiders” were placed in the inner receiving chamber with no other animal present for 5 min, but no shocks were administered. The day after Interim, the 5‐day Testing Phase 2 began, which was implemented with procedures identical to Testing Phase 1.

### 2.5 Dividing observers into “Natural Avoiders” and “Testing 1 Non‐avoiders”

As described above, after Testing 1, the animals in each cohort used for behavioral experiments were divided into two groups based on their tendency to avoid the dark chamber during Testing 1. “Natural Avoiders” were those animals that exhibited the greatest tendency within a cohort to avoid the dark chamber, while “Testing 1 Non‐avoiders” were those animals within a cohort that showed little or no evidence of avoidance. Additional information about how animals were assigned to the “Natural Avoiders” and “Testing 1 Non‐avoiders” groups is provided in the Supplementary Methods section of the Supporting Information.

### 2.6 Behavioral coding

All videos were assessed by one of three human observers according to the criteria described in the Supplementary Methods section of the Supporting Information. In order to ensure observers scored videos consistently, all three human observers had to watch and code at least three of the same videos three times. The human observers only proceeded to coding other experimental videos if they had at least 95% scoring consistency with themselves and with other raters.

### 2.7 Statistical analysis of behavior

Preferences for the dark chamber were assessed using repeated‐measures ANOVAs with dark chamber preference as the dependent variable. Independent variables were Testing Phase (within‐subject factor, coding for Baseline, Testing 1, or Testing 2 as appropriate) and Experimental group (between‐subject factor coding for Control Observer or Testing Observer in Testing 1, and Control, “Natural Avoider”, or “Testing 1 Non‐avoiders” in Testing 2). A covariate of non‐interest indicating the cohort of the Observer was included in all analyses. Significant interactions were interpreted via planned comparisons between testing phases or planned comparisons between groups. Since each repeated measurement within an experimental phase was not independent from the others, multivariate tests were used to interpret the results of all within‐subject effects. In all analyses, “Δ dark preference Testing 1” is the cumulative average dark chamber preference during Testing 1 (5 days) subtracted from the cumulative average dark chamber preference during Baseline (5 days). “Δ dark preference Testing 2” is the cumulative average dark chamber preference during Testing 2 (5 days) subtracted from the cumulative average dark chamber preference during Baseline (5 days). Intersubjectcive Avoidance, or “IA” is computed as the inverse of the dark chamber preference on a single day of testing subtracted from the cumulative average dark chamber preference during Baseline.

Relationships between IA and grooming, rearing, and social investigation in the electrophysiology experiments were assessed using Spearman's rank correlations, linear regression, and quadratic regression.

### 2.8 Immunohistochemistry

**Figure 3:**
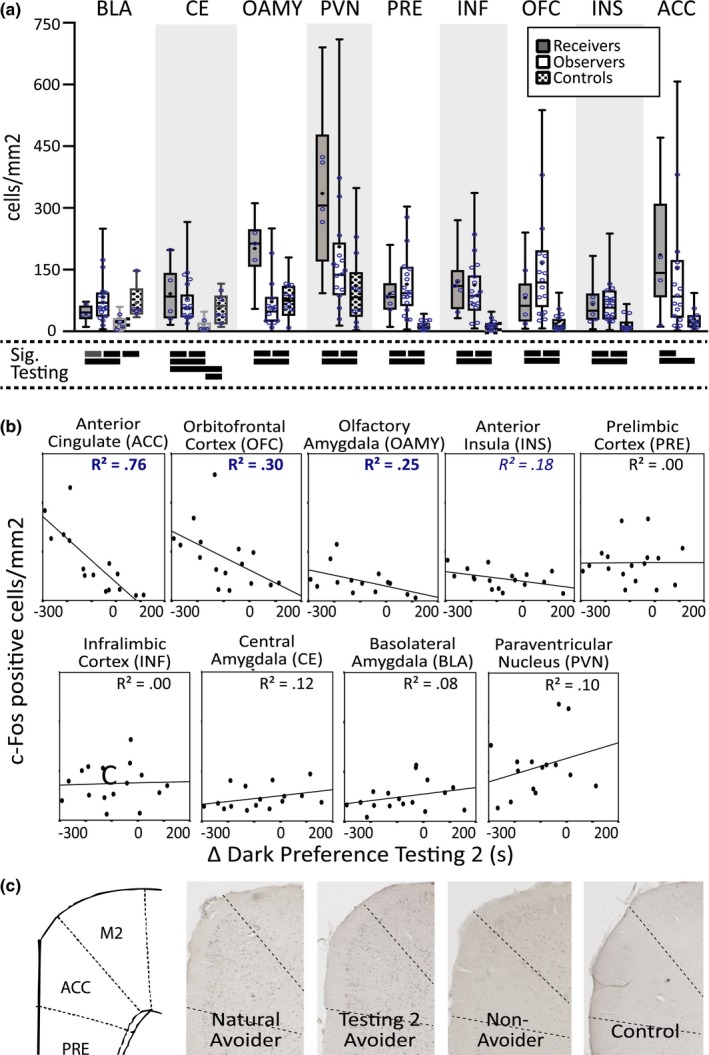
Common and unique elevated c‐Fos expression patterns in Receivers and Observers compared to Controls. (a) c‐fos expression in Observers, Receivers, and Controls. Boxes indicate middle 50% of the data. The line represents the median of the data. Whiskers represent the maximum and minimum values. Blue circles indicate the median values for each rat in the analysis. Significance testing symbols underneath the plot depict the results of pairwise comparisons. Black lines indicate a significant difference between groups, *p *=< .05 when Bonferroni multiple‐comparison corrections are applied. Gray lines indicate a significant difference between groups, *p *=< .05, when the multiple comparisons correction is not applied. Controls who naturally preferred the light and controls who naturally preferred the dark were combined unless their means were significantly different (in which case they are depicted with separate light and dark gray boxes, respectively, in the chart). BLA, basolateral amygdala; CE, central amygdala; OAMY, olfactory amygdala; ACC, anterior cingulate; PVN, paraventricular nucleus; PRE, prelimbic cortex; INF, infralimbic cortex; OFC, orbitofrontal cortex; INS, anterior insula; ACC, anterior cingulate. (b) Δ Dark Preference Testing 2 plotted against density of c‐Fos expression (untransformed data shown; statistics used transformed data). Blue r^2^ values in bold are significant, *p* < .05. Blue r^2^ values in italics approach significance, *p* < .08. (c) Examples of c‐Fos expression in the ACC of a Natural Avoider, Testing 2 Avoider, Non‐avoider (never demonstrated IA), and a Control Observer from approximately +2.75 mm anterior of Bregma. The corresponding atlas slice is illustrated on the left; the approximate boundaries of the ACC are represented by dashed lines

Details of the immunohistochemistry procedures are provided in the Supplementary Methods section of the Supporting Information. Given practical constraints on how many rats could be run in the appropriate circadian window of one day, the brains of 19 of the 33 Observer rats run in the IA test were collected (selected with an effort to collect brains from Observers with a wide range of behavior). In addition, brains were also collected from five Receivers, six control animals who naturally preferred the dark, and four control animals who naturally preferred the light. Five of the Observers and two of the Receivers had compromised brain tissue in 1 or more of the brain regions tested and were excluded from a subset of analyses. All analyses in Figure 3 have at least three animals in each experimental group.

### 2.9 Microscopy, image processing, and cell counting

Digital images of all brain structures of interest were taken and saved with the same illumination settings. A rat brain atlas was used to define the coordinates and boundaries of the brain structures analyzed (Paxinos & Watson, 2007). The coronal limits of brain regions of interest and the procedures used to automate cell counting are provided in the Supplementary Methods section of the Supporting Information. A minimum of four sections across each area of interest were counted for each rat, and at least three rats were included in each experimental group (ie: Receivers, Observers, or Controls). The Observer experimental control groups always had at least 14 rats. Controls that naturally preferred the dark and controls that naturally preferred the light were combined into one group unless a planned comparison indicated that they were significantly different from one another (see Statistics section).

### 2.10 Statistical analysis of c‐Fos immunohistochemistry

Outliers were identified using Tukey's hinges (>25th or 75th percentiles ± 1.5 interquartile range) and were winsorized (replaced with Tukey's hinges ± 1.5 interquartile range) before any of the c‐Fos data between groups were compared. Data were also examined for skewness and kurtosis, and either transformed to their logarithms (for the mixed‐effects model) or square‐roots (for the regressions) to improve normality. The means of c‐Fos immunoreactivity between experimental groups were compared using a mixed‐effects model with a fixed effect for experimental group and random effects specific to every rat to accommodate unequal amounts of repeated measures (see the Supplementary Methods section of the Supporting Information for details of model) (Gueorguieva & Krystal, 2004). The result of the mixed‐effects model was used to test all pairwise hypotheses between groups. When the means of the controls that naturally preferred the light and controls that naturally preferred the dark were not significantly different, they were combined. The mixed‐effects model were performed on log‐transformed c‐Fos variables, but the plots in Figure 3a of the main text depict raw data. *p*‐values with and without a Bonferroni multiple comparisons correction are reported.

Correlations and regressions testing relationships between mean c‐Fos activity and IA test performance (Tables 2 and 3) were performed using Pearson correlations and hierarchical regression. Hierarchical regressions were performed to determine the unique relationship of individual c‐Fos variables on behavior when other c‐Fos variables were accounted for. Since these analyses only examined Observers, square‐root transformations were sufficient to meet normality and homogeneity of variance requirements and were applied to the ACC, CEN, OAMY, and PVN data, but not required for the other five brain regions in this analysis. Statistics were performed on square‐root transformed c‐Fos variables (Tables 2 and 3, *R* ^2^ values in Figure 3b), but the plots in Figure 3b of the main text depict raw data. In all correlation analyses, a *p*‐value =< .05 (without multiple comparison corrections) was accepted as statistically significant, though marginally significant results (*p *=< .08) were also reported.

### 2.11 Local field potential data collection and pre‐processing

#### 2.11.1 Surgery

**Figure 4:**
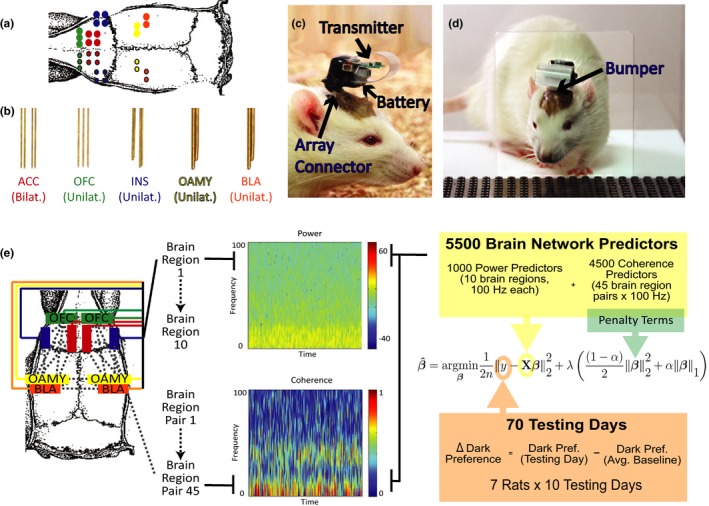
Surgical and Statistical Design. (a) Target location of individual electrode wires in each electrode bundle for the simultaneous recordings in the anterior cingulate (ACC), orbitofrontal cortex (OFC), anterior insula (INS), olfactory amygdala (OAMY), and basolateral amygdala (BLA). Colors indicate bundles of electrodes in same brain region. (b) Configurations of electrode bundles custom designed for each brain region. (c) Configuration of wireless headstage. (d) Observers pass easily through the door of the testing apparatus with the wireless headstage on. (e) Schematic of Elastic Net Analysis: 5500 characteristics of brain network activity are used to model behavior in ten days of testing in the Intersubjective Avoidance (IA) test (*N *= 7)

Ten microwire electrode bundles were implanted in ten 8–9 week‐old rats in the brain areas coordinates described in Table 1. Each bundle was custom‐designed to have the configuration depicted in Figure 4a and 4b in order to ensure all the bundles could be implanted simultaneously. All bundles were created using previous published procedures (Dzirasa, Fuentes, Kumar, Potes, & Nicolelis, 2011). In addition to the bundles, six stainless‐steel screws were inserted into the skull for stability. A ground wire was wrapped around a screw over the cerebellum. To ensure rats could safely be pair‐housed after surgery, (1) the completed implant was kept very close to the skull with a low profile, (2) the omnetics microconnector was carefully placed towards the back of the head facing the rest of the body so that it could not easily be reached by another rat, and (3) dental cement was shaped carefully around the connector and the wires to make it difficult for another rat to chew or grab any part of the implant (Figure 4c, d). After full recovery from surgery, rats were returned to their home cage with their cagemate for at least one week before experiments begun. Habituation for the experiments began when animals were about 10 weeks old. Three animals ultimately had to be excluded due to damaged omnetics microconnectors (caused by interactions with cagemates). After completion of the study, all electrode placements were confirmed in the remaining seven rats with nissl staining.

**Table 1:** Locations and descriptions of microwire electrode bundles

Brain region	A/P coordinate	M/L coordinate	D/V coordinate	No. Wires
L/R ACC	2.5	±0.5	1.6	4/4
L/R OFC	3.7	±2.0	3.8	3/3
L/R INS	2.2	±4.0	4.5	4/4
L/R BLA	−3	±5.0	7.5	2/2
L/R OAMY	−1.4	±3.2	8.8	2/2

ACC, anterior cingulate; OAMY, olfactory amygdala; OFC, orbitofrontal cortex; INS, anterior insula; BLA, basolateral amygdala.

#### 2.11.2 Acquisition parameters and procedures

All recordings were collected continuously using a custom‐made 31‐channel wireless headstage from Triangle BioSystems International (http://www.trianglebiosystems.com/). LFPs were sampled at 2000 Hz, preamplified (800×), notch‐filtered online to remove electrical artifacts, low‐pass filtered (250 Hz), and digitized at 2000 Hz using NeuroWare© software (Triangle BioSystems International). Overhead videos were collected throughout all recording sessions. Random patterns of square waves were sent simultaneously to the analog input of the recording system and a LED located at the edge of the video field of view to be able to precisely align behavioral events with neural recordings offline.

#### 2.11.3 Local field potential oscillatory power

A sliding‐window Fourier transform was applied to the LFP signal using a 1‐s window with no overlap. These 1‐s windows were aggregated and averaged for subsequent analyses.

#### 2.11.4 Local field potential cross‐structural coherence

Coherence was calculated using the Matlab (MathWorks) *mscohere* function. Coherence “is a function of the power spectral densities, *P*_xx_(*f*) and *P*_yy_(*f*), of x and y, and the cross power spectral density, *P*_xy_(*f*), of x and y: *C*_xy_(*f*) = (| *P*_xy_(*f*)|^2^)/ *P*_xx_(*f*) *P*_yy_(*f*).” A sliding window of 1 s with a 1‐s step and no overlap was used; transform parameters were chosen to allow for a frequency resolution of 1 Hz. These 1‐s windows were aggregated and averaged as appropriate for subsequent analyses.

#### 2.11.5 Note about lower local field potential frequencies

The primary analyses implemented in this study rely on knowing whether an Observer is in the dark or light chamber. Given that animals can move from the light to the dark chamber in less than a second, we felt it was imperative to have a minimum time‐resolution of one second. The trade‐off for this time resolution, however, is that fewer cycles of low frequency oscillations are captured in each window. Thus, the results from very low frequency ranges (especially <4 Hz) should be interpreted with more caution than results from higher frequency ranges.

#### 2.11.6 Local field potential amplitude correlation and phase analysis

All amplitude and phase analyses were restricted to time periods when Observers were in the dark chamber. Similar to previously published methods (Kumar et al., 2014; Likhtik, Stujenske, Topiwala, Harris, & Gordon, 2014), LFP data was filtered using butterworth bandpass filters designed to isolate LFP oscillations in 1 Hz bins, and then the instantaneous phase of the filtered data was computed using the Hilbert transform. To compare the relationship between the phase fluctuations of two signals from separate sources, the instantaneous phase difference (ϕ_Region1_ – ϕ_Region2_) was calculated for each time point, and the mean resultant length (MRL) of the entire phase difference time series from the dark periods of each experimental day was determined. These MRL values could range from 1 to 0. MRL values of 1 indicated that the phases of the two signals were perfectly synchronized (distributions of their phase differences clustered around a single angle/phase). MRL values of 0 indicated that the two signals were not synchronized at all (the distributions of phase differences were uniform across all angles/phases). Importantly, all phase relationships measured in this way were independent of the amplitude of the signals being compared (Tass et al., 1998).

#### 2.11.7 Aggregating data across electrodes

The power, coherence, or MRL values calculated from all correctly‐placed electrodes with reliable data (e.g. signal did not drift dramatically or saturate during movement) within a brain region or between pairs of brain regions were averaged before subsequent analyses.

#### 2.11.8 Imputation

There were some missing data points in our electrophysiology data matrix due to misplaced electrodes, excessively noisy individual electrodes, 1 session when the recording computer malfunctioned, and 2 sessions when an Observer spent the entire session in the light chamber. Since ENET models cannot accommodate missing data, we imputed the electrophysiological data to fill in these missing values before running all ENET models. A “donor” sample of the same size as the number of observations that did not have missing data was created by selecting vectors from all observations that did not have missing data randomly with replacement. A donor vector was chosen from this donor sample for each observation with a missing value, randomly with replacement. This procedure was repeated 10 times.

### 2.12 Regularized linear regression using Elastic Net penalty

The principles and motivation for the Elastic Net framework are discussed in the Supplementary Methods section of the Supporting Information.

#### 2.12.1 Model

Let ***y*** represent the vector of 70 IA responses (7 rats × 10 testing days for each rat), or the vector of residual IA responses once IA is regressed on changes in dark chamber grooming and social interaction. These responses represent Observers’ change in dark chamber preference in seconds on each day of Testing compared to their mean preference during the five days of Baseline. For rat *i*, the vectorx ***x***_*i*_ contains the measurement for its *p *= 5500 neural predictors. These neural predictors include (1) the change in power in 1 Hz oscillation bands from 1 to 100 Hz of each of the ACC, OFC, INS, BLA, and OAMY from baseline to each testing day (1000 predictors when all electrodes in a brain region are averaged) and (2) *either* the magnitude of the change in coherence, the change in amplitude correlation, or the change in MRL in 1 Hz oscillation bands from 1 to 100 Hz in each possible pair of these brain regions from baseline to each testing day. The neural activity for the seven rats (or six rats when the data from one rat is left out) is represented by matrix *X* with the dimensions *n* × *p*, where *n* = 70 due to the 10 testing days for every rat. Let β represent the coefficients when ***y*** is regressed on neural predictors. Usual least squares or maximum likelihood estimates of these coefficients cannot be calculated due to the *high‐dimensionality* problem. Thus, we impose the ENET penalty on β. ENET estimates the regularized regression coefficients βenet as a solution of the following optimization problem:

(1) $$\beta_{\mathrm{enet}}=\underset{\beta }{\text{argmin}}\left\{\sum_{i=1}^{n}{\left(y_{i}-\beta_{0}-\sum_{j=1}^{p}x_{ij}\beta_{j}\right)}^{2}+\lambda \sum_{j=1}^{p}\left(\alpha \beta_{j}^{2}+\left(1-\alpha \right)\left|\beta_{j}\right|\right)\right\},$$

where α and λ are tuning parameters that are sected using 10‐fold cross‐validation.

#### 2.12.2 Procedure

For the ENET‐*Dark* analyses, the *X* matrix was based on time points when individual Observer rats were in the dark chamber. For the ENET‐*Light* analyses, the *X* matrix was based on time points when individual Observer rats were in the light chamber. All ENET estimates were computed using the Matlab *Lasso* function. A 10‐fold cross validation procedure was used to choose the optimal values of α and λ that minimized mean squared error in predicting IA (the chosen α and λ values had the smallest mean squared error when all 10 cross‐validation sets were averaged). We ran the 10‐fold cross validation procedure on ten separate imputations of missing data in the *X* matrix. The reported coefficients represent the median coefficient from all ten imputations (each of which had α and λ values chosen by their own 10‐fold cross validation procedure). Power and coherence/amplitude correlation/MRL parameters were always modeled jointly.

#### 2.12.3 Leave‐one‐rat‐out analyses

To assess the consistency of our ENET procedure, we repeated the ENET‐*Dark* analyses with single animals removed from a model. We assessed the consistency of the predictors retained in these models compared to the full ENET model by (1) visualizing the number of times a parameter had a nonzero coefficient in the ENET results of each of the seven *Leave‐one‐rat‐out* analyses, and (2) calculating the true positive (TP), true negative (TN), false positive (FP), and false negative (FN) rates across of the ENET‐*Leave‐one‐rat‐out* analyses. We assessed the consistency of the coefficient magnitudes across models by computing a root mean squared error (RMSE) for LFP parameters in the TP, FP, and FN categories of predictors (Hastie, Tibshirani, & Friedman, 2009). We also used the *leave‐one‐rat‐out* analyses to estimate the predictive performance of our ENET‐procedure according to the median absolute error (MAE) fraction, or fraction of error reduced by using the ENET model compared to the null model when predicting the concatenated IA values for all seven rats. The procedures used to implement these analyses are detailed in the Supplementary Methods section of the Supporting Information.

## 3 Results

### 3.1 The intersubjective avoidance test

The IA test measures the extent to which a rat will take action to avoid another rat in distress. The IA test achieves this through intentionally biasing an Observer rat's physical location by providing the Observers with an opportunity to avoid bright light. The relative strength of an Observer's active aversion to another rat's pain – or “Intersubjective Avoidance” – is then measured relative to its initial location bias. In other words, the IA test pits intersubjective avoidance against light avoidance to assess the extent of negative affect caused by witnessing another rat's distress.

The IA test's primary measure of interest is how long the Observer spends in one of two outer chambers, both of which are separated from an single inner chamber by a transparent wall with holes (Preobrazhenskaya & Simonov, 1970). Rats naturally find bright light aversive, as illustrated by the fact that they will press a lever to terminate light stimuli at a rate that correlates with the light's intensity (Campbell & Messing, 1969; Keller, 1941). Taking advantage of this aversion, we kept outer chamber 1 dimly‐lit and outer chamber 2 brightly‐illuminated, which we hypothesized would induce an avoidance of outer chamber 2 under normal conditions. Each daily session (5 min), one “Receiver” was placed in the inner chamber, while one “Observer” rat was placed in outer chamber 2 and allowed to run freely between outer chambers 1 and 2 (see Figure 1a). No Receiver‐Observer pairs were cagemates, and rats were matched so that no Receiver‐Observer pair was repeated during testing.

During Baseline (5 days), Observers and Receivers were left undisturbed during the 5‐min daily sessions. As predicted, Observers exhibited “Light Avoidance”, or LA, during Baseline (mean dark preference = 58 ± 77 (SD) seconds, significantly greater than zero in a one‐way repeated‐measures ANOVA with cohort as a covariate, *F*_1,31_ = 6.74, *p *=* *.01, Figure 1b “Baseline” panel), and preferred the dark outer chamber all 5 days.

To assess rats’ response to other rats’ pain, we implemented Testing 1 (5 days). During Testing 1, all conditions were kept the same as Baseline except now Receivers received electrical shocks whenever an Observer entered the dark chamber (three shocks every 10 seconds; shocks continued until the Observer exited). We hypothesized that if Observers disliked witnessing a Receiver's distress more than they disliked bright light, they should choose to reduce their light avoidance—and perhaps avoid the dark chamber all together—during Testing 1. We found that, indeed, as a group Observers perceived Receivers’ distress to be equally aversive to bright light, because “Testing Observers” (*N* = 33 spread across 4 cohorts) significantly decreased their LA during Testing 1 (*F*_1,26_ = 6.77, *p *=* *.02 in a repeated‐measures ANOVA with cohort as a covariate; Figure 1b,c). Within this general pattern of Testing Observers, many individual Observers had extreme reactions and found witnessing other rats get shocked *more* aversive than (as opposed to equally aversive to) being exposed to bright light (Movie S1 and Figure 1d). These Observers demonstrated innate “Intersubjective Avoidance” (“IA”) by avoiding the dark chamber, instead of the light chamber, during Testing 1. Overall, Testing Observers’ change in LA contrasted with Control Observers (*N* = 10) who were exposed to baseline conditions for the duration of the experiment and who maintained their LA throughout Testing 1 (significant interaction indicated that Testing and Control Observers’ LA differed during Testing 1, but not during Baseline, *F*_1,35_ = 8.91, *p *<* *.01 in a repeated‐measures ANOVA with cohort as a covariate; Figure 1b).

Visual inspection of the IA test videos suggested that Observers’ avoidance behavior was strongly tied to the Receivers’ distress. However, to ensure that Observers’ IA (or avoidance of witnessing other rats get shocked) was not a nonspecific locomotor response due to increased general arousal or distress rather than aversion, we ran a control experiment in which Receivers were shocked when Observers entered the light chamber instead of the dark chamber during Testing (*N *= 6 in each group). Under these conditions, Observers’ LA significantly *increased* rather than decreased (*F*_1,5_ = 55.30, *p *<* *.01 in a repeated‐measures ANOVA with cohort as a covariate, Figure 1e). Thus, the direction of change in Testing Observers’ chamber preference during the IA test is specific to the location of a Receiver's distress, confirming that Observers find exposure to Receivers’ distress aversive, not just generally arousing. Observer rats’ decisions about where to move were tightly tied to how their actions related to the experiences of the Receiver rat.

### 3.2 Personal experience increases IA

Our experiments designed to find the neural correlates of IA would benefit from Observers’ IA being as strong as possible. We hypothesized that Observers’ IA would increase after having experienced foot shock themselves, similar to how humans report more empathy for harms they have personally experienced (Barnett, Tetreault, & Masbad, 1987; Eklund, Andersson‐Straberg, & Hansen, 2009), and similar to how rats open a door to let another rat escape a pool of water more quickly if they have previously been exposed to the pool of water themselves (Sato, Tan, Tate, & Okada, 2015). We capitalized on Observers’ performance variability during Testing 1 to test this. Observers who demonstrated little or no IA during Testing 1 were designated “Testing 1 Non‐avoiders” (*N *= 18; see Methods for details about how groups were chosen). We tested whether experience with foot shock would increase their IA during an Interim Phase (1 day; Figure 2a). After Testing 1, these animals were placed in the Receivers’ chamber with no other rat present, and shocked with three shocks every thirty seconds for the duration of the 5‐min Interim Phase. Starting the next day, Testing 2 commenced (5 days, identical to Testing 1).

We also tested whether rats that showed strong or intermediate IA in Testing 1, designated “Natural Avoiders” (*N *= 15), would continue to show IA in Testing 2. Since their IA was often already close to ceiling (as indicated by some Observers spending most of each Testing session in the light chamber), we tested whether their natural IA would persist over time without being shocked during the Interim Phase.

Indeed, despite never experiencing shock themselves, Natural Avoiders exhibited stronger IA than LA throughout Testing 1 and Testing 2 (Natural Avoiders vs. Controls *p *<* *.01 for both Testing 1 and 2, planned comparison; Figure 2b–d). These rats were consistently and persistently more motivated to avoid Receivers’ distress cues than they were to avoid innately aversive bright light.

Testing 1 Non‐avoiders had similar behavior to Controls during Testing 1 (Testing 1 Non‐avoiders vs. Controls: *p *=* *.17). However, as a group they increased their IA (reduced their LA) steadily each day of Testing 2 (Testing 1 Non‐avoiders vs. Controls: *p *=* *.04, Figure 2b–d). The fact that the group of Testing 1 Non‐avoiders’ increased their IA in Testing 2 compared to Testing 1 illustrates that experiencing shock oneself usually enhanced rats’ negative subjective experience of witnessing shock to Receivers. We exploited this observation in subsequent electrophysiology experiments by subjecting all rats to shocks during the Interim Phase.

### 3.3 Anatomical localization of IA

Having established a behavioral paradigm that elicited and maximized IA in rats, we next implemented a strategy to determine the neural mechanisms that manifest IA. Since the brain regions involved in IA test performance were completely unknown, the first step of our approach was to use c‐Fos (an immediate early gene used as an indicator of neuronal activity; Clayton, 2000) immunoreactivity to screen for brain regions that might be involved. We examined nine brain areas chosen for their known involvement in self‐reported human empathy/social behavior or negative emotion in humans and rodents: anterior cingulate (ACC), anterior insula (INS), orbitofrontal cortex (OFC), infralimbic cortex (INF), prelimbic cortex (PRE), paraventricular nucleus of the hypothalamus (PVN), olfactory amygdala (OAMY), central nucleus of the amygdala (CE), and basolateral nucleus of the amygdala (BLA) (Kim et al., 2015; Rilling & Sanfey, 2011; Stowers, Cameron, & Keller, 2013). The relationships between IA and c‐Fos immunoreactivity in these nine areas were examined in brains harvested one hour after testing on the last day of Testing 2 (Figure 3, Table 2).

**Table 2:** Pearson correlations between Testing Observers’ IA test performance and c‐Fos immunoreactivity

Intersubjective avoidance	Intersubjective avoidance			
	Average IA (Testing 1)	Average IA (Testing 2)	Average IA (Testing 1 + 2)	IA on Perfusion Day
Average IA (Testing 1)	–	–	–	–
Average IA (Testing 2)	0.51*	–	–	–
Average IA (Testing 1 + 2)	0.81**	0.92**	–	–
IA on Perfusion Day only	0.58**	0.89**	0.90**	–
**Brain region**				
Anterior Cingulate (“ACC”, *N *= 15)	−0.36	−0.87**	−0.77**	−0.69**
Olfactory Amygdala (“OAMY”, *N *= 14)	−0.31	−0.55*	−0.49***	−0.27
Orbitofrontal Cortex (“OFC”, *N *= 16)	0.01	−0.5*	−0.34	−0.26
Anterior Insula (“INS”, *N *= 17)	−0.06	−0.43***	−0.35	−0.35
Central Amygdala (“CE”, *N *= 16)	0.01	0.34	0.28	0.44
Paraventricular Nucleus (“PVN”, *N *= 15)	0.26	0.31	0.34	0.30
Basolateral Amygdala (“BLA”, *N *= 17)	−0.19	0.28	0.14	0.36
Infralimbic Cortex (“INF”, *N *= 16)	−0.25	0.05	−0.06	0.27
Prelimbic Cortex (“PRE”, *N *= 17)	−0.09	0	−0.02	0.19

The values in the table represent the Pearson correlation between the entity in the column header and the entity in the row header. Natural Avoiders, Testing 2 Avoiders (who avoided during Testing 2 but not Testing 1), Non‐avoiders, and Inverse Responders (who spent *more* time in the dark chamber during Testing) were included in these analyses. *Δ Dark Preference Testing 1 *=* * average dark preference across 5 days of Testing 1 – average dark preference across 5 days of Baseline. *Δ Dark Preference Testing 2 *=* * average dark preference across 5 days of Testing 2 – average dark preference across 5 days of Baseline. *Δ Dark Preference Testing 1 + 2 *=* * average dark preference across 10 days of Testing 1 and 2 – average dark preference across 5 days of Baseline. *Δ Dark Preference Perfusion Day *= dark preference on day of perfusion ‐ average dark preference across 5 days of Baseline.

**p <* *.01, *p <* *.05, ***p <* *.08.

All regions had elevated c‐Fos immunoreactivity in the Receivers compared to Controls (Figure 3a). Most brain regions also had elevated c‐Fos immunoreactivity in the Observers compared to Controls (Observers’ c‐Fos immunoreactivity in the BLA and CE was greater than the Controls who preferred the Light, but not the Controls who preferred the Dark; the OAMY was the only brain region with elevated c‐Fos immunoreactivity in the Controls compared to Observers), consistent with human neuroimaging studies showing overlapping but distinct patterns of hemodynamic activity when humans receive versus observe pain (Bernhardt & Singer, 2012). Observing and receiving distress also invoked distinct patterns of neural activity. CE, OAMY, PVN, INF, and ACC activity was higher in Receivers than Observers, while BLA, PRE, OFC, and INS activity was (sometimes slightly) higher in Observers than Receivers.

More instructive, of the nine brain regions that had elevated c‐Fos immunoreactivity in Observers compared to Controls, only three correlated with individual differences in Observers’ IA: ACC, OFC, and OAMY (Figure 3b, 3c, Table 2). The INS also approached significance (*p *=* *.08). Remarkably, the ACC could account for 76% of the variance in individual rats’ IA in Testing 2, 59% in Testing 1 + 2, and 48% on the day of perfusion (Table 2). Furthermore, sequential hierarchical regressions that incorporate brain regions into regression models one at a time indicated that the ACC was the only brain region that could account for unique IA variance above and beyond the effects of the other brain regions tested (Table 3). These results demonstrate anatomical selectivity; although almost all brain regions had elevated c‐Fos immunoreactivity in Observers compared to controls, only three of those brain regions (or four, if considering the correlations that approached significance in the insula) had c‐Fos immunoreactivity that correlated with individual differences in IA performance. Having identified the ACC, OFC, OAMY, and INS as IA‐encoding brain regions using c‐Fos, next we determined how these brain regions interacted while rats made intersubjective decisions.

**Table 3:** Hierarchical regressions assessing relationship between c‐Fos expression and IA during Testing 2

	SE	β	ΔR^2^
**ACC c‐fos entered first**			
Step 1: ACC c‐fos	5.27	−0.89	0.79**
*Model 1*			
Step 2: OAMY c‐fos	2.11	−0.10	0.01
Step 3: OFC c‐fos	0.47	−0.19	0.03
*Model 2*			
Step 2: OFC c‐fos	0.45	−0.20	0.03
Step 3: OAMY c‐fos	2.10	−0.06	<0.01
**Cortical c‐fos entered first**			
Step 1: OAMY c‐fos	3.14	−0.55	0.31*
*Model 1*			
Step 2: ACC c‐fos	6.46	−.837	0.49**
Step 3: OFC c‐fos	0.47	−0.19	0.03
*Model 2*			
Step 2: OFC c‐fos	0.77	−0.40	0.14
Step 3: ACC c‐fos	6.60	−0.77	0.38**
**OFC c‐fos entered first**			
Step 1: OFC c‐fos	0.76	−0.56	0.31*
*Model 1*			
Step 2: ACC c‐fos	5.64	−0.80	0.51**
Step 3: OAMY c‐fos	2.10	−0.06	<0.01
*Model 2*			
Step 2: OAMY c‐fos	3.18	−0.40	0.14
Step 3: ACC c‐fos	6.60	−0.77	0.38**

ACC, anterior cingulate; OAMY, olfactory amygdala; OFC, orbitofrontal cortex. Since the highest correlations found in Table 2 were with IA averaged across Testing 2, Average IA (Testing 2) was used as the dependent variable in these hierarchical regressions. c‐Fos activity in the ACC, OAMY, and OFC were entered as step‐wise independent variables. ΔR2 represents the variance in IA that can be accounted for by the c‐fos in the brain region of that step *after* the variance accounted for in previous steps is removed. SE and β represent the standard error and beta, respectively, associated with the regression model at that step. The ACC and OAMY variables were square‐root transformed to improve normality. All collected testing Observer brains were included in these analyses.

**p <* *.01, *p <* *.05.

### 3.4 The elastic net strategy for identifying brain oscillations that encode IA

Since decision‐making and social processing requires multiple brain regions (Barrett & Satpute, 2013; O'Connell & Hofmann, 2011; Ruff & Fehr, 2014; Stanley & Adolphs, 2013), and many of the brain regions that contribute to social decision‐making can have multiple different functions, we hypothesized that the functions the ACC, OFC, OAMY, and INS execute during an intersubjective decision would be based on not only the activity within each brain region, but also how that activity was coordinated with activity occurring in other brain regions (McIntosh, 1999; O'Connell & Hofmann, 2011; Park & Friston, 2013; Pessoa, 2014; Teles et al., 2015). Local Field Potentials are powerful tools for measuring behaviorally‐relevant coordinated brain activity (Akam & Kullmann, 2014; Buschman & Kastner, 2015; Henry et al., 2014; Voytek & Knight, 2015). By analyzing different frequencies of LFPs from many brain regions simultaneously, in theory, it is possible to combine calculations of LFP power and LFP coherence to examine neural activity *within* brain regions at the same time as examining information passed *between* brain regions. This information can then be used to infer the structure of spatially‐distributed functional neural networks with good temporal and spatial resolution.

To examine how brain regions work together to encode intersubjective decisions, we recorded LFPs bilaterally from all of the brain regions whose c‐Fos immunoreactivity predicted differences in individual Observers’ IA while rats performed the IA test. The regions recorded from included the ACC, OFC, OAMY, and INS; we also recorded LFPs from the bilateral BLA due to a previous report of BLA LFP activity in observational fear conditioning (Jeon et al., 2010). In total, 30 channels of LFPs spread across all 10 brain regions (the aforementioned five brain regions bilaterally) were collected wirelessly and simultaneously from a cohort of seven rats with a custom‐designed headstage (Figure 4a,b).

Our unique surgical strategy allowed us to record LFPs simultaneously from 10 spatially separated brain regions, but it also posed new statistical challenges not posed by studies that examine only one or two brain regions. If we examined the relationship between each LFP predictor and IA independently, we would have to ignore the established relationships within bands of LFP predictors and would encounter challenges with multiple comparison corrections that are overly‐conservative in situations where independence is violated (Johnson et al., 2010). If we examined the relationship between all of the LFP predictors and IA in one model, the relationship between predictors would be taken into account, but we would encounter the *high‐dimensionality* and *high‐correlation* problems (see the Supplementary Methods section of the Supporting Information for a more information about these problems). We used an elastic net (ENET) regularized regression strategy borrowed from the machine learning field (Zou & Hastie, 2005) to address these challenges. Our ENET procedure modeled the relationship between IA (7 rats across 10 Testing days, for a total of 70 behavioral data points) and (1) the power in 1 Hz‐wide oscillation bands from 1 to 100 Hz averaged across all the electrodes implanted in each of the ACC, OFC, INS, BLA, and OAMY (1000 predictors when all electrodes in a brain region are averaged) and (2) the magnitude of coherence in 1 Hz oscillation bands from 1 to 100 Hz in each possible pair of these brain regions (4500 predictors when all electrodes in a brain region are averaged) (Figure 4e). In doing so, the ENET framework permitted us to infer the frequency composition and boundaries of oscillation bands relevant to IA in a joint, data‐driven fashion, even though such oscillations were highly correlated and far more oscillations were measured than behavioral data points (see the Supplementary Methods section of the Supporting Information for a full discussion).

By taking advantage of the elastic net framework, we were able to examine simultaneously in one statistically valid model the relationship between IA and all 5500 neural predictors that represented frequency‐specific oscillations within *and* between ten brain regions. If coherence predictors were retained in the ENET solution, it would indicate that oscillatory relationships between brain regions accounted for unique variance in IA and were required to optimally describe behavior. The ENET framework thereby provided us with a strategy for inferring what “neural context”, if any, was related to empathy's influence on rats’ intersubjective judgments and behaviors, despite the large number of neural predictors compared to behavioral data points.

Of note, the ENET regularization framework works by estimating models using many combinations of possible penalty parameters, and by applying cross validation procedures to identify the penalty parameters that minimize the mean squared error in predicting a dependent variable. Since the number of included predictors is dynamic in this procedure, no strategy has yet been developed to characterize the degrees of freedom in an ENET model, which in turn means that there is also no currently accepted method for applying traditional p‐values to ENET models (Lockhart, Taylor, Tibshirani, & Tibshirani, 2014). Despite these differences from traditional nonpenalized statistical methods, all predictors retained in an ENET solution are considered statistically interpretable.

### 3.5 Oscillations within and between brain regions are required to optimally encode IA

**Figure 5:**
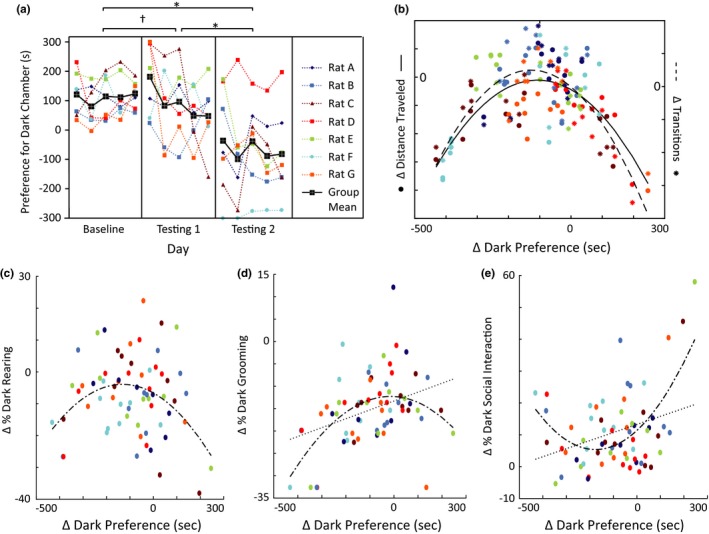
Behavior of Electrophysiology Cohort. (a) Observers implanted with microwires show Intersubjective Avoidance (IA). * *p *=* *.02. † *p *=* *.08. (b, c) The total distance traveled (circles), number of transitions made by observers (stars), and Δ % Dark Rearing were correlated in an inverse U‐shaped curve with IA (quadratic fits *p *<* *.01; linear fits were not significant). (d, e) IA had quadratic relationships with Δ % Dark Grooming and Δ % Dark Social Investigation (*p *<* *.01), as well as linear relationships with Δ % Dark Grooming (Pearson correlation = −0.37, *p *<* *.01; Spearman's rho = −0.36, *p *<* *.01) and Δ % Dark Social Investigation (Pearson correlation = −0.32, *p *<* *.01; Spearman's rho not significant). The data points associated which each rat are color‐coded in each panel

We acquired LFP recordings during all days of Baseline and Testing of the IA test. All seven Observers we recorded from were given experience with shock during the Interim phase to maximize IA during Testing 2. Neither the surgical nor recording procedures dramatically interfered with IA test performance; six of the seven rats reduced their dark preference during Testing 1 compared to Baseline and the cohort's overall IA approached significance during Testing 1 (*p *=* *0.08) and achieved significance during Testing 2 (*p *=* *.02; Figure 5a). Thus, IA was induced in our electrophysiological cohort.

Unless otherwise specified, the results we describe are from models that use *change in dark preference* on one testing day compared to the average dark preference during Baseline as the dependent variable, and the *change in electrophysiology measures* averaged across that testing day compared to those averaged across 5 days of Baseline as predictors (see Methods and Materials for details). This is a critical point, because all rats demonstrated light avoidance during Baseline. Therefore, the behavioral measure used in our statistical model assessed the relative strength of each rat's intersubjective avoidance compared to their light avoidance, and the neural predictors used in our statistical model represented neural activity that differed from time periods when rats were exhibiting light avoidance. As such, the neural features identified by our analysis should reflect the networks that are preferentially engaged or disengaged during intersubjective avoidance, rather than those equally engaged by all types of avoidance, in general.

**Figure 6:**
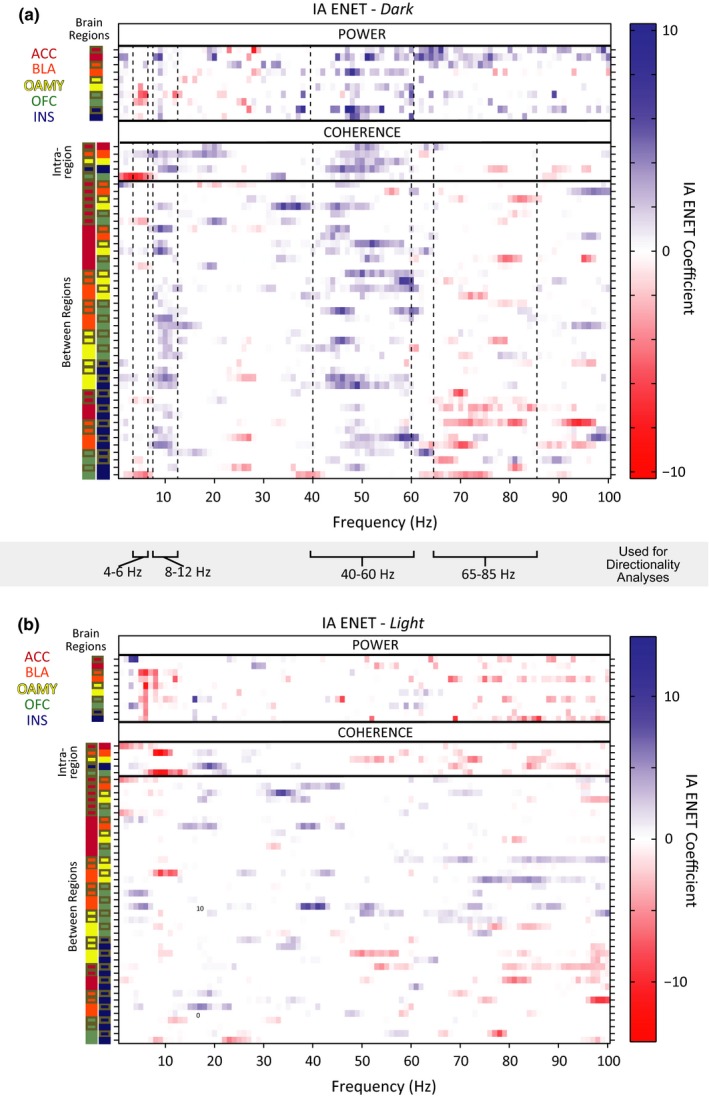
Network Oscillations predict individual differences in Intersubjective Avoidance (IA). (a) ENET‐*Dark* coefficients. Intersubjective avoidance was regressed on local field potential (LFP) activity averaged across only time points when Observers were in the dark chamber, and the elastic‐net framework was applied to regularize (or penalize) the regression coefficients. (b) ENET‐*Light* coefficients. Intersubjective avoidance was regressed on LFP activity averaged across only time points when Observers were in the light chamber, and the elastic‐net framework was applied to regularize (or penalize) the regression coefficients. For (a and b) all parameters were modeled jointly, brain regions pairs are indicated by color‐coded squares, and no pairs are repeated. Left hemisphere regions are outlined in gray, right hemisphere regions have no outline

We hypothesized that the neural responses that would be most relevant to an Observer rat's intersubjective decisions would be those elicited when the Observer was witnessing a Receiver get shocked, because the averseness of the experience is presumably what motivates the observed IA. Thus, we began by applying the ENET framework to a penalized regression that regressed IA (defined as the change in dark preference for each Testing day compared to Baseline) on LFP activity averaged across only time points when Observers were in the dark chamber (therefore only when Observers were witnessing Receivers get shocked). Across all analyses, our ENET procedure did not select one sole oscillation predictor within a highly correlated band (which would suggest over‐sparsity) or assign all neural predictors with coefficients (which would suggest under‐sparsity) (Figure 6a).

Critically, we found that the ENET solution contained *both* power and coherence parameters (Figure 6a). This result indicates that power *and* coherence measurements were required to optimally describe the IA of the rats in this study. In other words, the relationship of activity between brain regions carried unique information about how a rat will decide to respond to another rat's pain, above and beyond signals from any set of brain regions in isolation.

Visual inspection of the ENET‐*Dark* analysis illustrated distributed IA neural networks characterized by three data‐driven frequency bands of oscillations whose predictors correlated with IA in the same direction (Figure 6a): 4–6 Hz (“theta”, although typical theta bands are defined as 4–8 Hz), 8–12 Hz (alpha), and 40–60 Hz (low gamma). Theta oscillations correlated negatively with IA. These theta oscillations were observed almost exclusively within the OFC (power predictors), or between the OFC and the ACC (coherence predictors). In stark contrast, alpha power correlated positively with IA and was observed widely across almost all brain regions tested. The alpha oscillations were observed as power predictors within almost all brain regions tested, and were observed as coherence predictors between brain regions primarily when an amygdalar region was involved (and not observed often as coherence predictors between brain regions when an amygdalar region was not involved). Interestingly, IA‐signaling low gamma oscillations were even more prevalent than alpha oscillations, and were observed both within and between most brain regions tested.

IA‐signaling 65–85 Hz (high gamma) oscillations had a very different pattern than any of the other oscillatory bands observed. Some high gamma oscillatory power was observed within the ACC that correlated positively with IA. Almost all other high gamma oscillations correlated negatively with IA, were between brain regions, and were dominated by synchrony with the INS. This was notable given that alpha power and low gamma power within the INS, as well as alpha and low gamma coherence between the INS and other brain regions, correlated positively with IA. These results indicated that the INS might be involved in more than one opposing contributions to intersubjective decisions.

Visual inspection of the ENET‐*Dark* analysis revealed some predictors in 12–40 Hz and 85–100 Hz ranges in addition to the ranges described above, but the patterns of predictors in the in 12–40 Hz and 85–100 Hz ranges were much more sparse and did not necessarily correlate with IA in the same direction (Figure 6a).

Importantly, IA‐encoding oscillations were largely absent when Observers were in the light chamber, except for a band of theta power in all regions but the ACC that correlated negatively with IA (ENET‐*Light*; Figure 6b). Thus, the oscillatory responses that signaled IA were different from those elicited by bright light, were temporally specific, and occurred in response to witnessing another rat get shocked, not while avoidance was underway.

The ENET framework implements “feature selection” and “feature estimation” simultaneously. We used a leave‐one‐rat‐out cross‐validation analysis to assess the reliability of each of these aspects of the ENET solution respectively. The ENET framework's feature selection was not driven by a single animal, because the features of the ENET results were similar when single animals were removed from a model (SI Figure 1). Across all cross‐validation sets (where the data from one rat was removed in each set), the predictors that had zero coefficients matched approximately 90% of the time (see 2 for details of how this percentage is calculated). The median true negative rate (median absolute deviation), or “TN”, was 0.90 (0.05). The predictors that had nonzero coefficients matched approximately 62% of the time; the median true positive rate (median absolute deviation), or “TP”, was 0.69 (0.06). Within these, the predictors that had coefficients with absolute values greater than 6, 5, 4, 3, 2, or 1 in the ENET‐*Dark* analysis had nonzero coefficients in the cross‐validation sets approximately 98%, 96%, 94%, 90%, 85%, and 78% of the time (SI Table 1; the Supplementary Methods section of the Supporting Information for details of how these rates were computed). Therefore, neural predictors with large coefficients in the primary ENET‐*Dark* analysis (having absolute values of >3) had nonzero coefficients in almost every reduced model as well. Although there was an overall median false negative (FN) rate of 0.31 (0.06) and false positive (FP) rate of 0.10 (0.04) across the cross‐validation sets, the mean and medians of the absolute value of the magnitudes of such unmatched coefficients were all <1 (SI Table 2). Therefore, predictors that were not consistently retained across the ENET‐*Dark* and ENET‐*Leave‐One‐Rat‐Out* analyses had very small coefficients. The overall mean root mean squared error and standard deviation across the cross‐validation sets was 1.26 (0.14) for TPs, 0.98 (0.22) for FNs, and 0.99 (0.11) for FPs, where the scale of the error is on the same scale as the coefficients. This indicates that the magnitudes of the coefficients retained in the models across the cross validation sets were relatively consistent. Put together, these analyses indicate that neural predictors with small coefficients in the primary ENET‐*Dark* analysis should be interpreted with more caution, but overall the features retained in the ENET results were stable and reliable, and the details of the predictors with large coefficients could be interrogated further to discern candidate brain network dynamics that encode IA.

The magnitudes of the weights applied to each predictor retained in the ENET solution were more sensitive to specific animals than the identity of the predictors retained in the model. Overall, the ENET solution fit the seventy behavioral data points moderately well, but systematically underestimated very low and high values of IA, most of which were performed by rats C, D, and F (SI Figure 2a, 2c). As would be expected, then, the ENET model did a poor job of predicting the IA of those rats when they were left out of the training data (SI Figure 2b). In contrast, the ENET did a moderate to good job of predicting the IA of rats A, B, E, and G when they were left out of the training data. Put together, the MAE fraction of the ENET model using the leave‐one‐rat‐out analysis was 0.76 (where MAE fraction is the median predicted error relative to a null model that predicts the median IA value for every data point; see 2). This modest MAE fraction is comparable to the model fits reported by other attempts to predict behavior with neurological data in highly‐correlated high‐dimensional settings (Lu, Yang, Lin, Li, & Wei, 2013; Manolio et al., 2009; Wager, Atlas, Leotti, & Rilling, 2011), but indicates that the LFP predictors identified by our ENET analysis encode middle ranges of IA much better than extreme values of IA. Rats with extreme behavior may be recruiting brain regions outside of those recorded from in this study.

### 3.6 Relationships between IA‐signaling Oscillatory Networks and Related Behaviors

To assess whether IA‐encoding oscillations identified by the ENET‐*Dark* analysis could be explained or confounded by changes in other more detailed behaviors, we assessed the relationships between IA, locomotion, rearing, grooming, and social investigation. The relationships between IA and LFP activity discussed above are not likely fully explained by changes in locomotor activity or rearing, because IA had an inverse‐U shaped relationship with both locomotor activity and change in percent dark chamber rearing (*p *<* *.01 for distance and change in transitions; *p *=* *.01 for percent dark chamber rearing) and linear relationships between IA and these variables were not significant (Figure 5b,c). However, IA did have both linear and quadratic relationships with changes in percent dark chamber grooming and social investigation (Figure 5d,e). Overall, when Observers exhibited more IA (had greater reductions in time spent in the dark chamber), they spent smaller percentages of their time in the dark chamber engaged in social investigation (according to parametric correlations, but not nonparametric correlations; Pearson correlation(*r*) = −.32, *p *<* *.01; *p* for Spearman's rho (*r*_*s*_) = .19) or grooming compared to Baseline (*r *= −.37, *p *<* *.01; *r*_*s*_ = −.36, *p *<* *.01). Significant quadratic relationships also indicated that sometimes Observers with strong IA spent a greater percentage of time engaged in social investigation, and occasionally grooming, as well (Figure 5d, e). Thus, the LFP measurements used the ENET – *Dark* analysis were confounded by systematic changes in the amounts of social investigation and grooming. Visual investigation of the relationships between changes in social investigation/grooming and LFP activity suggest that some of the ENET – *Dark* results might indeed be influenced by differences in social investigation/grooming, especially coherence in the lower frequencies or high gamma frequencies (SI Figures 5, 4). If true, these relationships would be challenging to disentangle, given that social investigation and grooming have linear relationships with IA in the same direction, quadratic relationships with IA in opposite directions, and have correlations with each other that approach significance (Pearson coefficient = −0.23, *p *=* *.06). Further, changes in grooming and social investigation correlate with multiple EA‐encoding LFP predictors in opposite directions, despite their first order linear correlation with EA is in the same direction (see, in particular, 8–18 Hz coherence that correlates positively with changes in grooming but negatively with changes in social investigation; SI Figures 3, 4). Overlapping relationships of this kind are known to create suppressor effects and coefficient flipping in regression analysis (Friedman & Wall, 2005; Ganzach, 1997; Julious & Mullee, 1994), but have not been addressed in p≫n settings.

To gain insight into whether the linear relationships between changes in social investigation/grooming and LFP activity might explain some of the relationships observed between IA and LFP activity, we regressed IA on changes in percentages of dark chamber social investigation and grooming to remove the variance in IA that could be accounted for by social investigation and grooming. We then repeated the ENET procedure with the residuals from this analysis as the dependent variable. The results replicated many of the LFP relationships identified in our previous analyses, although the coefficients for most of the predictors were smaller (SI Figure 5), and the MAE fraction increased to 0.89 (larger numbers indicate poorer model fit). In addition, although the general patterns of LFP parameters in the alpha, theta, low gamma, and high gamma bands remain the same, the precise boundaries of these bands sometimes shifted in the IA‐Residuals ENET‐*Dark* analysis compared to the IA ENET‐*Dark* analysis in the regions where social investigation and grooming correlated with IA in opposite directions (see the alpha coherence between the INS or OFC and other brain regions for examples). Thus, changes in social investigation and grooming contribute and interact with some of the patterns observed between IA and LFP activity, but they likely do not account for all the observed relationships.

**Figure 7:**
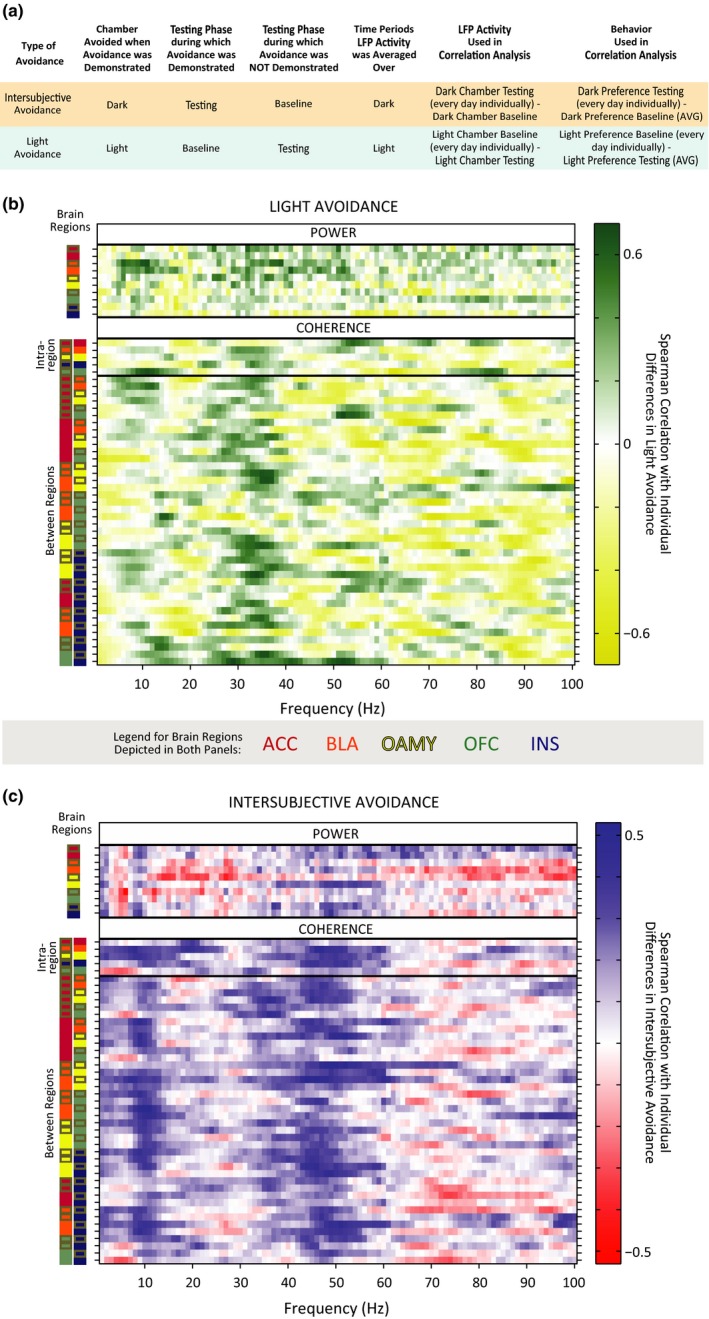
Exploratory Analysis: Intersubjective Avoidance is encoded by different oscillations than Light Avoidance. (a) Details of how Intersubjective Avoidance (IA), Light Avoidance (LA), and their corresponding neural predictors were defined and calculated. (b) Spearman correlation coefficients between all 5500 local field potential (LFP) predictors and individual differences in LA (35 behavioral data points dues to 7 Observers × 5 days of Baseline). (c) Spearman correlation coefficients between all 5500 LFP predictors and individual differences in IA (blue to red). 30–40 Hz oscillations are mostly specific to LA while 45–60 Hz oscillations are mostly specific to IA. There is modest overlap in the 8–12 Hz oscillations that correlate with LA and IA (more so in power predictors than coherence predictors)

As another exploratory investigation, we ran a correlation analysis to examine how IA‐encoding oscillations might compare to LA (light avoidance)‐encoding oscillations (Figure 7a). Spearman Rank coefficients were computed between neural predictors while Observers were in the light chamber during Baseline and LA (Figure 7b), and a separate set of Spearman Rank coefficients were computed between neural predictors while Observers were in the dark chamber during Testing and IA (Figure 7c). The ENET framework could not be applied reliably to the LA models due to the fact that we had half as many behavioral data points during Baseline as we did during Testing, so the individual Spearman Rank coefficients we present should not be interpreted strongly. Nonetheless, this exploratory analysis makes it clear that while some alpha power oscillations might be common to both types of avoidance, many of the alpha coherence oscillations and almost all of the low gamma oscillations are unique to IA. In contrast, it appears that LA might be preferentially encoded by beta (25–40 Hz) oscillations. These exploratory findings will need to be replicated and confirmed, but they support an interpretation that rat intersubjective decisions are encoded by specific frequencies of oscillations between brain regions that have multiple functions within emotional processing, social cognition, and decision‐making, and that the frequencies of those oscillations likely determine the selective role those brain regions play during intersubjective decision‐making.

### 3.7 Phase synchronization: a mechanism for the relationship between oscillatory networks and IA

The first set of ENET results indicated that synchrony between brain regions can have unique functional relevance distinct from—and sometimes even in opposite directions from—activity within participating brain regions. Next, we wanted to determine which aspects of between‐region synchrony could best explain its correlation with IA. The long‐range oscillatory synchronization reflected by LFP coherence can be affected by two different types of synchrony (Canolty et al., 2010; Wang, 2010). Amplitude correlation occurs when the magnitude of specific frequencies of LFP power are systematically related. Phase coherence occurs when the peaks and valleys of specific frequencies of LFPs align. Amplitude correlation and phase coherence can occur simultaneously, but they can also occur independently (Siegel, Donner, & Engel, 2012; Srinath & Ray, 2014; Womelsdorf et al., 2007). In order to determine whether phase coherence, on its own, would relate to individual differences in IA, we bandpass filtered individual 1‐Hz frequency bins of each recorded LFP signal and computed their instantaneous phase. Phase coherence was measured by computing the mean resultant length (MRL) of the instantaneous phases of two LFP signals while an observer was in the dark chamber on a given day; a MRL value of 0 indicated that the phases of the two signals were completely uncorrelated and random, while an MRL value of 1 indicated that the phases of the two signals were perfectly in sync. We then repeated the ENET‐*Dark* analyses retaining all of the original LFP power values, but replacing coherence values with MRL values (ENET – *Dark MRL*). This analysis allowed us to determine whether IA was encoded by the phase relationships of specific frequencies of oscillations between brain regions.

**Figure 8:**
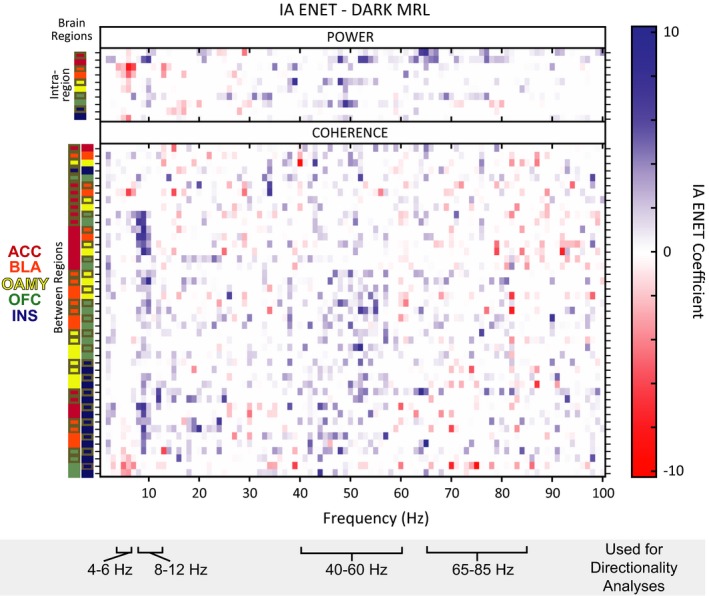
Oscillatory phase synchrony predicts individual differences in Intersubjective Avoidance (IA). ENET‐*Dark MRL*. The predictors in this ENET analysis included the same power predictors as the ENET‐*Dark* analysis, but the coherence predictors were replaced with mean resultant length (MRL) phase synchrony measurements on each testing day compared to the baseline average. All parameters were modeled jointly. Brain regions pairs are indicated by the color‐coded squares; no pairs are repeated. Left hemisphere regions are outlined in gray, right hemisphere regions have no outline. The bands depicted in the gray box, chosen by visual inspection of the ENET‐*Dark* and the ENET‐*Dark MRL* results, were analyzed for directionality

We found that MRL values signal IA through patterns that are similar to those observed with coherence (Figure 8a), even when the residuals of IA regressed on grooming and social interaction are used (SI Figure 6). These results indicate that the individual differences in IA measured in this study could be at least partially explained by how well the phase relationships of oscillations in a distributed network that spans the ACC, INS, OFC, BLA, and OAMY are temporally aligned.

### 3.8 Directionality of IA‐signaling network oscillations

The ENET analyses suggested that different bands of coherence between brain regions encoded moderate values of IA in different ways. To gain greater insight into what functions might be encoded by those coherence bands, we designed a directionality analysis to determine whether the phases of IA‐encoding oscillations in one brain region reliably lead or followed the phases of the same frequency of oscillations in other brain regions (Kumar et al., 2014; Likhtik et al., 2014). We focused this analysis on the frequency bands and brain pairs whose coherence signaled IA in the ENET‐*Dark* analyses. Directionality was computed for the time points when an Observer was in the dark chamber for all the predictors retained in the ENET‐*Dark* solution, which roughly fell into the four data‐derived, functionally distinct bands discussed in the “*Oscillations Within and Between Brain Regions are Required to Optimally Encode IA*” section: theta (4–6 Hz), alpha (8–12 Hz), low gamma (40–60 Hz), and high gamma (65–85 Hz). These analyses allowed us to determine whether phase‐related signals in one brain region of each of these IA‐signaling frequency bands led or lagged those in another brain region, and if so, by how much.

**Figure 9:**
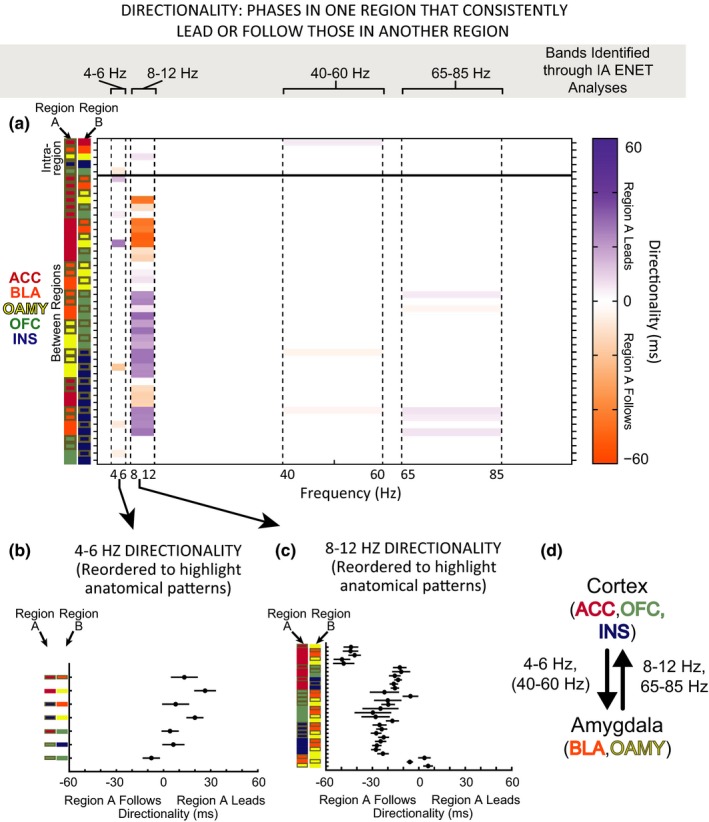
Directionality of Intersubjective Avoidance (IA)‐predictive phase oscillations. (a) Directionality of each of the indicated pairs in the order they are labeled (e.g. a negative directionality value for R BLA × R OFC indicates that basolateral amygdala [BLA] oscillations follow, or lag behind, orbitofrontal cortex [OFC] values). Directionality values with z‐scores whose absolute value is <1.96 (the 95% confidence interval) are not shown. The values represented by color in this panel are shown with more detail on the x‐axis of panels b and c. (b) Nonzero directionality values and confidence intervals for coherence predictors in the theta band of the ENET‐*Dark* analysis that surpassed the z‐score cut‐off. (c) Nonzero directionality values and confidence intervals for coherence predictors in the alpha band of the ENET‐*Dark* analysis that surpassed the z‐score cut‐off. Although the values depicted with color in panel a are depicted on the x axes of panel b and panel c, the brain pairs are re‐organized in panel b and panel c to better illustrate the anatomical patterns discussed in the text. (d) Summary schematic of the directionality analysis results

Strikingly, when directionality was present in theta‐like frequencies, it was predominantly from the cortical regions (ACC, INS, and OFC) to amygdalar regions (BLA and NLOT) (Figure 9a, b, d). In contrast, directionality in alpha frequencies was always in the opposite direction, from amygdala to cortical regions (Figure 9a, c, d). In addition, alpha signals in amygdalar regions preceded those in INS and OFC, which in turn preceded those in the ACC. Little directionality was observed in low or high gamma bands, but when it was observed, insular activity preceded amygdalar regions (low gamma), or amygdalar regions preceded primarily the insula (high gamma).

## 4 Discussion

Intersubjective decisions are the result of many interacting cognitive processes that collaborate to ultimately manifest actions that reduce others’ pain (Barrett & Satpute, 2013; Betti & Aglioti, 2016). We have provided evidence that frequency‐specific oscillations between spatially distributed brain regions involved in social cognition and negative emotion may serve as a mechanism by which some of these cognitive processes are coordinated. This evidence was made possible through an interdisciplinary experimental approach that incorporated a new behavioral paradigm to assess intersubjective avoidance (IA) in rats, c‐Fos mapping to identify brain regions likely to be involved in IA, and the adaptation of penalized regression methods for multisite LFP recordings measured from ten spatially distributed brain regions.

The behavior we describe in this study demonstrates that witnessing another rat in distress is aversive to observing rats. These observations add to a growing literature showing that nonhuman species make intersubjective decisions (Bartal, Decety, & Mason, 2011; Bartal, Rodgers, Sarria, Decety, & Mason, 2014; Jeon et al., 2010; Langford et al., 2006; Panksepp & Lahvis, 2011; Sato et al., 2015), and advance previous rodent behavioral studies by illustrating that rat intersubjective decisions cannot be explained solely by generalized arousal. While this growing literature is exciting, it is important for the field to continue developing new tasks that can provide deeper insight into how similar rodent intersubjective decisions are to humans’ social decisions. In particular, although rat IA is likely to be related to some aspects of human empathy, at present it is unclear whether the IA test described here is useful for studying altruistic intentions or conscious understanding of another's pain. The IA test does not examine what motives Observers have when they demonstrate IA, and therefore cannot determine whether Observers’ IA is primarily self‐interested (perhaps to reduce shared fear, for example), and it remains unknown whether Observer rats can consciously understand other rats’ pain. Be that as it may, the present study does demonstrate that the IA test is useful for studying “negative intersubjectivity”, or the phenomenon of having a negative subjective experience when another individual is having a negative experience. Negative intersubjectivity is a consistent predictor of human prosocial behavior and a strong inhibitor of violent behavior, regardless of whether an human actor consciously understands the pain other people are going through (Schaich Borg, 2016). In addition, negative intersubjectivity is a developmental antecedent to more sophisticated moral behaviors in humans, and may ultimately contribute to phenomena like altruistic intentions or the recognition of moral content (Pfaff & Sherman, 2015; de Waal, 2012). Thus, understanding how the brain encodes decisions motivated by negative intersubjectivity may provide avenues for studying the neural mechanisms underlying human decisions to act in ways that alleviate others’ pain; future research will hopefully provide more insight into how such decisions relate to different conceptions of “empathy” (Schaich Borg, 2016).

The brain regions we recorded LFPs from in this study were selected through c‐Fos assays. c‐Fos immunoreactivity was elevated in Observers in most of the brain regions examined. This result confirms that Receivers’ distress is very salient to Observers, and is consistent with the interpretation that Receivers’ distress is aversive. Despite this general elevation across social cognition brain areas, only c‐Fos immunoreactivity in the ACC, OFC, and OAMY (and marginally the INS) correlated with individual differences in IA. The c‐Fos results are strikingly similar to neuroimaging studies reporting that humans’ self‐reported empathy for another person correlates linearly with ACC activity and INS activity evoked by observing that person in pain (Bernhardt & Singer, 2012). The c‐Fos results are also consistent with neuroimaging studies implicating the amygdala and OFC in empathic processing (Betti & Aglioti, 2016). Overall, c‐Fos expression in the brain areas previously shown to be involved in human empathy seem to selectively signal individual tendencies toward IA in rats.

Once the bilateral ACC, OFC, OAMY, and INS were identified as candidate brain regions through c‐Fos analyses, our surgical strategy allowed us to examine LFP signals bilaterally in these regions (in addition to the BLA) while rats performed intersubjective decisions. For the first time in rats, these recordings provided a broad view of LFP networks in the brain that are related to intersubjective decisions. Plots of the independent correlations between LFP and IA illustrated that multiple bands of power and coherence LFP predictors correlated with Observer Rats’ IA (Figure 7c). The ENET framework allowed us to infer in a data‐driven fashion the relationship between LFP parameters and IA when the strong relationships between power and coherence predictors were taken into account. The leave‐one‐rat out analyses demonstrated that the LFP features retained in the ENET solutions were robust across cross‐validation sets. The magnitudes of the ENET coefficients were less resilient to differences in training data in these analyses. While the ENET solution captured intermediate values of IA fairly well, it could not accurately predict extreme demonstrations of IA or lack of IA (SI Figure 2). When the animals spent almost all of a testing day in the light chamber or the dark chamber, the absolute values of IA patterns predicted by the ENET – *Dark* model were dramatically shifted upward or downward respectively.

One possible reason the ENET – *Dark* model did not predict strong IA accurately might be that very few LFP samples were collected from the dark chamber when animals were exhibiting strong IA, since by definition, animals were strongly avoiding the dark chamber. An interesting result that might give further insight into why the ENET – *Dark* model did not predict extreme IA well was that c‐Fos immunoreactivity in the present study correlated most strongly with average IA during Testing 2, rather than IA on the day of perfusion. For example, ACC c‐Fos accounted for 76% of the variance in individual rats’ IA in Testing 2, 59% in Testing 1 + 2, but only 48% of the variance in individual rats’ IA on the day of perfusion.

Put together, the ENET‐*Dark* and c‐Fos results suggest that while the regions recorded from in this study might be highly involved in intermediate levels of IA and initial demonstrations of behavior, other brain regions that we did not record from may become involved once the avoidance behavior is learned. Relevant observations have been made during studies of active avoidance where animals need to learn to move to a certain part of a testing arena in order to avoid receiving an electrical shock. Fear responses have a U‐shaped relationship with avoidance in these contexts; although fear is initially needed to motivate the avoidance response, fear responses to the conditioned stimuli decrease dramatically once consistent avoidance responses have been performed (Kamin, Brimer, & Black, 1963). Likewise, the cortical electrophysiological patterns of animals who have been well‐trained in an avoidance task are more similar to those evidenced during quiescent, deactivated states than highly arousing or fearful states (Castro‐Alamancos, 2004). Thus, the motivating systems that help establish avoidance behavior are not the same as those that maintain avoidance behavior (Kamin et al., 1963). One possible explanation for the poor prediction of extreme IA in the present study might be that the brain regions we recorded from are involved in motivating intersubjective avoidance, but other brain regions become involved once the avoidance is fully learned and established. This could also account for why c‐Fos immunoreactivity in the present study correlated most strongly with average IA during Testing rather than IA on the day of perfusion. c‐Fos is not expressed during all kinds of neural activity (Clayton, 2000), and c‐Fos expression is often reduced or completely absent once a task has been mastered or fully learned (Bertaina‐Anglade, Tramu, & Destrade, 2000). Many of the Natural Avoiders in the c‐Fos experiments consistently exhibited IA throughout the last days of testing, so they may have fully learned the contingencies of the IA test which would in turn alter their c‐Fos expression during the final Testing days. Brain regions that are important for initiating avoidance even after it has been learned include the basal ganglia and substantia nigra *pars reticulata* (Hormigo, Vega‐Flores, & Castro‐Alamancos, 2016). It would be interesting to determine in future studies whether IA on perfusion day correlates better with c‐Fos in these areas than with c‐Fos in the ACC.

It is also important to acknowledge that while strongly negative IA was rarely observed in this study, Rat D in the electrophysiology cohort (Figure 5a) and approximately three animals in the purely behavioral cohorts (Figure 2d) did spend dramatically *more* time in the dark chamber after they were given experience with shock during the interim phase, rather than less time. These animals were often visibly distressed when they were removed from the testing apparatus at the end of each testing day, and visual inspection of their videos indicated that they spent a lot of their time in the dark chamber during Testing huddled in a corner. In contrast, observers that showed extremely positive IA tended to be calmer when they were removed from the testing apparatus. This general pattern is consistent with what has been observed in traditional avoidance training; animals who are not able to learn avoidance contingencies exhibit much more fear than those who do. Furthermore, animals who are extremely fearful due to previous conditioning are sometimes very poor at learning how to avoid the stimulus that is causing the fear (Weiss, Krieckhaus, & Conte, 1968). This raises the possibility that the neural systems that led to a few Observers spending more time in the dark chamber during Testing may be different from those that initially motivated IA for most rats. The neural systems that contributed to animals huddling in the corner of the dark chamber may be more directly involved in freezing responses, and therefore may preferentially involve the paraventricular nucleus, central amygdala, or bed nucleus of the stria terminalis (Tovote, Fadok, & Lüthi, 2015). We speculate that one way to improve behavioral predictions for rats who show very poor IA in the IA test would be to incorporate measurements of neural activity from these brain regions into the models described in the present study. Additional experiments would be needed to test this hypothesis, but the results of the c‐Fos and LFP analyses taken together underline the importance of using multiple, complimentary measures of neural activity to dissect the biological basis of behavior, provide evidence that intersubjective decisions are the result of multiple information processing systems, and suggest that the LFP parameters identified in the present study are only a subset of those needed to describe the full range of behavior in the IAT.

Acknowledging that the ENET‐*Dark* results characterized intermediate values of IA better than extreme values, one of the important results of this study is that the ENET procedure repeatedly indicated that power *and* coherence neural parameters were required to optimally account for behavior when the LFP predictors were modeled jointly, even when single rats were left out of the analysis or when grooming and social investigation are taken into account. These results show that the aspects of IA captured by our ENET model is at least partially encoded by the relationship of activity between brain regions rather than by activity in single brain regions in isolation. These results indicate that neural context may be an important aspect of how the rodent brain encodes socially‐motivated actions, and that functions of individual brain areas during intersubjective decisions may be tailored by the activity occurring in other brain regions at the same time. The role of functional connectivity in vertebrate social behavior has been suggested through correlations of immediate early gene expression or cytochrome oxidase patterns after social tasks (Hoke, Ryan, & Wilczynski, 2005; Sakata, Coomber, Gonzalez‐Lima, & Crews, 2000; Teles et al., 2015; Yang & Wilczynski, 2007). Our results support those previous reports, and show with high temporal resolution in mammals that frequency‐specific mechanisms for coordinating electrical activity in spatially distributed brain regions, themselves, can encode individual differences in how rats integrate social distress information into their choices for action.

If specific frequencies of oscillations invoke distinct functions from brain regions, it might be possible to use multiple frequencies of oscillations to invoke multiple functions in the same brain region at the same time. Activity in the anterior insula synchronized with other brain regions at several distinct frequencies in this study, and those frequencies encoded IA in different ways. In particular, low gamma coherence between the INS and OFC/BLA correlated positively with IA, while high gamma coherence between the INS and OFC/BLA correlated negatively with IA. In addition, low gamma oscillations in the insula preceded those in the amygdala in the low gamma range, but followed those in the amygdala in the high gamma range. Although the direction of relationships of predictors in models with high collinearity should be interpreted with caution (Friedman & Wall, 2005; Ganzach, 1997; Julious & Mullee, 1994), these results are consistent with human neuroimaging studies suggesting the anterior insula has dynamic functional connections with multiple separate brain networks (Nomi et al., 2016), and may even orchestrate the switches between these networks (Menon & Uddin, 2010; Sridharan, Levitin, & Menon, 2008). These results are also consistent with the contradicting, paradoxical effects of insula lesions on addiction (Droutman, Read, & Bechara, 2015), and the paradoxical relationships between insula thickness and hemodynamic activity with psychopathy (Decety, Skelly, & Kiehl, 2013; Ly et al., 2012). A related observation has been made in humans, suggesting that functional connectivity during resting state between the INS, OFC, ACC, and amygdala correlates positively with “affective empathy” or the ability to share the emotional experiences of others, while functional connectivity between INS, bilateral superior temporal gyri/sulci, and brainstem at the same time correlates negatively with affective empathy (but positively with the ability to take the mental perspective of others) (Cox et al., 2012). Overall, specific frequencies of synchronization between neural populations in spatially distributed brain regions may permit different types of information transfer, which may in turn lead a brain region to have multiple computational functions within the same behavioral task.

One consequence of using oscillations between brain regions to encode behaviors is that activity within individual brain regions, when examined in isolation, can yield a fundamentally incomplete view of how the brain performs a function or behavior, and may even lead researchers to misinterpret necessity or sufficiency studies. For example, the opposing relationships of IA with low versus high gamma synchrony between INS and OFC/BLA would be completely occluded by lesion or sufficiency studies that manipulate all of the activity within the INS at once. Therefore, moving forward it will be important to continue examining neural context in the rodent brain in addition to isolated neural activity. Towards this end, the ENET computational framework and multisite recording strategies we describe here provide a straightforward method for inferring neural context that can be applied to future rodent studies that examine other brain regions or use other behavioral tests. Future studies may also benefit from incorporating computational modeling to understand the contributions of anatomical units whose functions are determined by multiple network interactions with multiple feedback mechanisms. Computational modeling may additionally help to untangle the confounding relationships between IA, grooming, and social interaction. Although our analysis using IA residuals suggest that the network patterns we report in the present study are not likely to be fully explained by grooming and social interaction, there are some interactions (especially in the alpha band), and it is unclear whether the experience of negative intersubjectivity, on its own, motivates changes in grooming and social interaction. More targeted analyses will be needed to comprehensively understand the nature of the relationships between these behaviors (as well as any other subsidiary behaviors we did not control for), IA, and individual LFP parameters. Especially since some of these behaviors may occur on time scales that are faster than the 1‐s windows we used to analyze our LFP signals, it will be useful for future studies to develop experimental preparations and statistical methods that can examine the contributions of individual types of movements to the overall IA measure we use in the present study.

To aid future studies designed to test how bands of LFPs in individual brain areas or pairs of brain areas encode intersubjective decision‐making, we propose the following working hypotheses about the cognitive subprocesses that might be supported by the specific oscillations we found to encode intermediate levels of IA in the present study. Locomotor decisions generally reflect cost‐benefit analyses of the positive and negative outcomes expected from salient stimuli in the environment (Hirayama et al., 2014). The alpha oscillations (8–12 Hz) we observed may contribute to the encoding of positive expected outcomes, as they are similar to those observed in the olfactory system of rats during positive social interactions (Tendler & Wagner, 2015). The theta (4–6 Hz) power and coherence oscillations we observed may contribute to the encoding of negative expected outcomes, as they are similar to those reported in olfactory regions during fear conditioning in rats (Tendler & Wagner, 2015), and in the ACC and BLA during observational fear conditioning in mice (Jeon et al., 2010). Alpha oscillations have also been reported in the hippocampus during motivated movement, while theta oscillations have been reported in the hippocampus reported during fear‐induced immobility (Oddie & Bland, 1998; Pineda, 2005; Sainsbury, 1998). We found alpha oscillations to be positively correlated with IA when Observers were in the dark chamber, and theta oscillations to be negatively correlated with IA when Observers were in either chamber. BLA theta oscillations, specifically, only correlated with IA when Observers were in the light chamber, and seemed to encode information passed from the cortex to the amygdala. The amygdala may be a key coordinator of the alpha signals, as the phase of its alpha oscillations preceded that of all other brain regions tested. When considered in light of previous studies, these results suggest that intersubjective judgments in the IA test might be the result of a conflict between appetitive social affiliation and learned fear, such that the outcome of a judgment is determined by the relative presence of alpha oscillations that mediate motivated social interaction and theta oscillations that mediate freezing responses. This interpretation is consistent with human studies showing that children who become overly distressed in response to witnessing another child's suffering are less likely than children who are only moderately distressed to help the suffering child in pain (Eisenberg, Eggum, & Di Giunta, 2010). In essence, in order to take action to reduce another's pain, we may need to suppress, or at least overshadow, our own fear circuits.

We found that the ACC may be a key player in the downstream output of the alpha signals, as the phases of its alpha oscillations followed those of all other brain regions tested. The alpha signals in the ACC might interact with the gamma signals in the ACC (Canolty & Knight, 2010). One potential explanation for the IA‐encoding gamma oscillations we observed comes from a recent study showing that mice missing the immediate early gene Arc (which has similar activity‐dependent properties to the immediate early gene c‐Fos used in the present study) have relatively reduced gamma oscillatory power during active tasks, but not at rest (Malkki et al., 2016). In addition, other studies that have shown that gamma oscillations correlate with performance during emotional memory tasks (Headley & Paré, 2013). These studies suggest gamma oscillations may facilitate the long‐term potentiation and depression associated with neuronal plasticity and learning. In the context of the IA test, gamma oscillations may integrate the binding of the affective responses mediated by the INS and AMY with value information in the OFC to potentiate action through the ACC and downstream regions such as the basal ganglia and substantia nigra *pars reticulata* (Hormigo et al., 2016; Shackman et al., 2011). When such action is potentiated through learning, intersubjective avoidance would occur with greater frequency and rapidity. An interesting hypothesis, then, is that the ACC may control the motor output of an intersubjective decision by integrating motivational information passed through alpha oscillations with learned motor responses encoded through gamma oscillations (Bosman, Lansink, & Pennartz, 2014). More experiments will be useful for testing this hypothesis, and for uncovering other potential computational roles of the oscillations identified in this study.

One of the primary motivations for trying to understand the neural basis of intersubjective judgement is to gain insight into how interventions could be designed to augment human prosocial behavior and decrease human violence. Not all humans would perform the intersubjective avoidance exhibited by the rats in this study, especially violent psychopaths. Even if we do not yet know a rats’ motives for demonstrating IA, understanding the neural mechanisms underlying rats’ decision to avoid other rats’ distress may provide insight into what neural mechanisms can be exploited to help humans avoid other humans’ distress as well, especially given that the brain regions we found to encode rat IA have also been implicated in human empathy and social processing. The results presented here suggest that it would be valuable to explore whether deep‐brain stimulation or transcranial magnetic stimulation targeting networks within the ACC, INS, OFC, and amygdala can be used to help treat pathologically antisocial human behavior, making sure to take the functional connectivity of the networks are taken into account (Smart, Tiruvadi, & Mayberg, 2015). Exploring such treatment options could ultimately help us reduce the influence of extremely violent individuals, but will also hopefully allow us to obtain a more mechanistic understanding of how one of the most complex, but fundamental, phenomena governing human societies works: the notion that humans make personal sacrifices to reduce others’ pain.

## Author Contributions

JSB conceived, performed, and analyzed all aspects of the behavioral and immunohistochemistry experiments. JSB also conceived the *in vivo* electrophysiological experiments, designed and performed the implantation surgeries, performed the *in vivo* electrophysiological experiments, preprocessed and prepared all electrophysiological data, conceived the ENET analysis strategy, codesigned and co‐implemented all ENET analyses and validations with SS and LL, and wrote the manuscript with SS, LL, DD, KD, and LdL. SS codesigned and co‐implemented the ENET leave‐one‐rat‐out validation analyses, performed the mixed‐effects analysis of the immunohistochemicy data, and wrote the manuscript with JSB, LL, DD, KD, and LdL. LL codesigned and co‐implemented the ENET analysis strategy, co‐implemented the ENET *leave‐one‐rat‐out* models, and wrote the manuscript with JSB, SS, DD, KD, and LdL. JH performed frame‐by‐frame behavioral coding of the video data from the *in vivo* electrophysiological experiments. DD provided statistical oversight for all ENET analyses of the *in vivo* electrophysiological experiments, and wrote the manuscript with JSB, SS, LL, KD, and LdL. KD jointly conceived the electrophysiology analysis strategy with JSB, oversaw all aspects of data collection and analysis for the *in vivo* electrophysiological experiments, and wrote the manuscript with JSB, SS, LL, DD, and LdL. LdL jointly conceived the behavioral test strategy with JSB, oversaw all aspects of data collection and analysis for the immunohistochemistry experiments and behavioral experiments that did not incorporate electrophysiology, and wrote the manuscript with JSB, SS, LL, DD, and KD.

## Conflict of Interest

The authors declare no competing financial interests.
