# Supplementary material for: Rat intersubjective decisions are encoded by frequency‐specific oscillatory contexts
Source: Brain Behav. 2017 May 5;7(6):e00710. doi: 10.1002/brb3.710 (PMC5474713; doi:10.1002/brb3.710)

­SUPPORTING INFORMATION

Rat intersubjective decisions are encoded by frequency-specific oscillatory contexts

Jana Schaich Borg^1,2,3^, Sanvesh Srivastava^4^, Lizhen Lin^5^, Joey Heffner^6^, David Dunson^7^, Kafui Dzirasa^2,8,9,10,11*^, and Luis de Lecea^3*^

**Affiliations:**

^1^ Social Science Research Institute, Duke University, Durham, North Carolina, 27710

^2^ Duke Institute for Brain Sciences, Duke University, Durham, North Carolina, 27710

^3^ Department of Psychiatry and Behavioral Sciences, Stanford University, Stanford, California, 94305

^4^ Department of Statistics and Actuarial Science, University of Iowa, Iowa City, Iowa, 52242

^5^ Department of Applied and Computational Mathematics and Statistics, University of Notre Dame, Notre Dame, Indiana, 78712

^6^ Department of Psychology, Cognitive, Linguistic & Psychological Sciences, Brown University, Providence, Rhode Island, 02912

^7^ Department of Statistical Science, Duke University, Durham, North Carolina, 27708

^8^ Department of Psychiatry and Behavioral Sciences, Duke University Medical Center, Durham, North Carolina 27710

^9^ Department of Neurobiology, Duke University Medical Center, Durham, North Carolina 27710

^10^ Department of Neurosurgery, Duke University Medical Center, Durham, North Carolina 27710

^11^ Dept. of Biomedical Engineering, Duke University, Durham, North Carolina, 27708

**Supplementary Methods.**

*Habituation procedures for the Intersubjective Avoidance (“IA”) Test*: Pilot experiments indicated that Observers’ dark preference was more consistent if they were habituated to the testing environment before data was collected. Habituation was performed by placing the Observers in the IA apparatus for five minutes with no Receivers present. Habituation days were repeated until the Observers anxiety and exploratory behaviors in the testing apparatus chamber stabilized, as determined by visual inspection of urination, fecal boli, general locomotor activity, and any visual demonstrations of distress (as described by [Whishaw and Kolb, 2005](#_ENREF_91); [Deacon, 2006](#_ENREF_23)). Two to five days were typically required to reach this point.

*Dividing observers into “Natural Avoiders” and “Testing 1 Non-Avoiders”*: Behavioral experiments involving each cohort were carried to completion before the next cohort was begun, and the goal was to end up with approximately equal numbers of "Natural Avoiders" and "Testing 1 Non-Avoiders" in each cohort to help reduce the statistical complications that would arise from confounding shock experience with cohort.  Raw preference for the dark chamber during Testing 1 (and the total amount of time a Receiver would be exposed to shocks), change in dark preference during Testing 1 compared to Baseline, and the time course of behavior over the five days of Testing 1 were all taken into account when assigning groups. Given that each cohort had a different distribution of behavior (Fig. 1C) and that our goal was to divide each cohort into roughly equal groups, these considerations had to be used to different extents in each cohort. As a result, occasionally Observers whose mean dark preference during Testing 1 was less than their mean dark preference during Baseline might still be assigned to the “Testing 1 Non-Avoiders” group, and Observers whose mean dark preference during Testing 1 was more than their mean dark preference during Baseline might still be assigned to the “Testing 1 Avoiders” group. For example, one Observer with a very strong Baseline dark preference reduced its dark preference mildly during days 1 and 2 of Testing (while still strongly preferring the dark chamber), but then resumed its original dark preference for the rest of Testing 1. Another Observer in the same cohort had a milder Baseline dark preference to start with, spent more time in the dark chamber for the first few days of Testing, but then preferred the light chamber by over two minutes by the last day of testing. Due to the amount of time the Receiver would be exposed to shocks in each case and the directions of the trends over the five days of testing, the first Observer was assigned to the Non-Avoiders group and the second Observer was assigned to the Avoiders group, even though their average dark preference across the five days of Testing 1 was less (in the case of the first Observer) and more (in the case of the second Observer) than their average dark preference during Baseline. This procedure accounts for the overlap of the two groups in the histogram shown in Fig. 2C; the overlap was due to the between-cohort differences in overall behavior.

*Criteria used for behavioral coding*: An animal was considered to be in a chamber when its body was at least three-fourths of the way through the door separating the two chambers. Each individual frame of the videos from the cohort used for electrophysiology were also coded for rearing, grooming, and social investigation. An animal was considered to be rearing when both front paws were completely off the ground (paws could be against a wall or the animal could be freestanding). An animal was considered to be grooming when it was attending to its own body and was engaged in a bout of characterized movements that included licking or rubbing its hands on its body. An Observer was considered to be engaged in social investigation when it was within an inch of the holes in the walls separating the inner and outer compartments, was oriented towards the Receiver, and was either sniffing or actively investigating in a directed fashion for at least 0.5 seconds.

*Immunohistochemistry* *procedures*. 1 hour after the last session of the experiment, rats were anesthetized with isoflurane and perfused transcardially with 200 mL PBS (pH 7.4) followed by 200 mL 4% paraformaldehyde in PBS. Brains were extracted, postfixed overnight in 4% paraformaldehyde at 4°C, put in 30% sucrose at 4°C until they sank, flash-frozen in isopentane, and then stored at -80°C. Each brain was sectioned into 5 separate series of 35 μm slices on a cryostat (Leica Microsystems) and stored in a solution of 30% glycerol, 30% ethylene glycol, and 40% PBS at -80°C until processing.

Free-floating sections were washed with PBS, incubated in 3% hydrogen peroxide solution, washed, blocked for 1 hour with 4% bovine serum albumin (BSA) in 0.2% PBST, incubated overnight in rabbit anti-c-Fos (1:8,000, Santa Cruz Biotechnology, Inc, K-25, sc-253) in the same blocking solution, washed, incubated in biotinylated goat anti-rabbit IgG secondary antibody (1:200, Vector Laboratories) for 1 h at room temperature, and washed again. Then the sections were incubated in an avidin-biotin ABC peroxidase solution (Vector Laboratories, #PK-6100) for 1 hour. Last, the sections were washed and stained using 3-3′diaminobenzidine-4 HCl (DAB) and nickel solution (Vector Laboratories, SK-4100). The resulting slices were mounted on Superfrost® Plus slides (Statlab Medical Products) and coverslipped with Permaslip (Alban Scientific).

*Coronal limits of brain regions analyzed in c-Fos analyses*:

| **Brain region** | **Anterior/Posterior boundaries relative to Bregma** |
| --- | --- |
| Anterior Cingulate (ACC, N=15) | 3.25 → 1.56 |
| Olfactory Amygdala (OAMY, N=14) | -0.84 → -1.44 |
| Orbitofrontal Cortex (OFC, N=16) | 5.16 →3.25 |
| Anterior Insula (INS, N=17) | 4.20 → 2.52 |
| Central Amygdala (CE, N=16) | -1.56 → -3.00 |
| Paraventricular Nucleus (PVN, N=15) | -1.08 → -1.56 |
| Basolateral Amygdala (BLA, N=17) | -1.72 → -3.36 |
| Infralimbic Cortex (INF, N=16) | 3.72 → 2.52 |
| Prelimbic Cortex (PRE, N=17) | 4.68 → 2.52 |

*Automated cell-counting procedures*: To count c-Fos positive cells in the PRE, OFC, INS, and ACC, whole-region mosaics of 10x photomicrographs were assembled using a computerized stage, a black-and-white CCD camera, and custom software from Steven Smith’s lab at Stanford University (http://code.google.com/p/smithlabsoftware/). To count c-Fos positive cells in the OAMY, CE, BLA and PVN, single 10x images from a Zeiss microscope (Axio Imager A.1) with a color CCD camera were sufficient to cover the brain region of interest. All pictures were coded with a unique identifier to obscure the group membership of each rat. Outlines of brain areas to be counted were hand drawn onto each saved picture and saved as a digital region of interest. Adapted ImageJ software (developed in collaboration with Ryan Luecke) was then used to normalize the background, threshold each picture according to a user-determined threshold, and count how many particles were present within each region of interest that passed the indicated threshold. Although many thresholds were experimented with to confirm that thresholding would not affect interpretations, for the data reported here, all images taken from the same microscope were thresholded at the same level. Some tissue was damaged during processing, and therefore could not be used for analysis. A minimum of four sections across each area of interest were counted for each rat, and at least three rats were included in each experimental group (ie: Receivers, Observers, or Controls). The Observer groups always had at least 14 rats. The final counts of rats and slices used in each experimental group were as follows:

| **Brain region** | **# Rats, *# Brain Slices* per Group** | | |
| --- | --- | --- | --- |
|  | **Observers** | **Receivers** | **Controls** |
| Anterior Cingulate (ACC) | 15, *225* | 3, *31* | 10, *208* |
| Olfactory Amygdala (OAMY) | 14, *59* | 3, *18* | 9, *37* |
| Orbitofrontal Cortex (OFC) | 16, *182* | 4, *42* | 4, *131* |
| Anterior Insula (INS) | 17, *244* | 5, *52* | 10, *158* |
| Central Amygdala (CE) | 16, *83* | 4, *20* | 4, *16* (dark)  5, *21* (light) |
| Paraventricular Nucleus (PVN) | 15, *112* | 4, *29* | 8, *53* |
| Basolateral Amygdala (BLA) | 17, *91* | 3, *14* | 4, *18* (dark)  4, *16* (light) |
| Infralimbic Cortex (INF) | 16, *166* | 4, *41* | 10, *120* |
| Prelimbic Cortex (PRE) | 17, *250* | 4, *58* | 10, *192* |

*Statistics:*

*ANOVA for comparing c-Fos immunoreactivity.* Data were log-transformed to their logarithms to improve normality before analysis, but the plots in Fig. 3A of the main text depict raw data. The following model was applied to the log-transformed c-Fos data:

$y_{ijk}= \alpha+ \mu_{i}+ b_{j}+ \epsilon_{ijk} , b_{j} \sim\mathrm{Normal}\left( 0,\sigma_{b}^{2} \right), \epsilon_{ijk} \sim Normal(0, \sigma^{2}$),

where α is the intercept, *µ_i_* is the mean effect of experimental group *i* (Controls, Observers, or Receivers*)*, *b_j_* is the block effect for rat *j* and follows a Gaussian distribution with mean 0 and variance $\sigma_{b}^{2}$ (this term accounts for the repeated measures associated with each rat), *k* represents the number of slices examined for each rat *j*, and $\epsilon_{ijk}$ is the residual error and follows a Gaussian distribution with mean 0 and variance $\sigma^{2}$. This ANOVA could accommodate the different numbers of brain slices available for each rat. The result of the ANOVA was used to test all pairwise hypotheses between groups. When the means of the controls that naturally preferred the light and controls that naturally preferred the dark were not significantly different, they were combined. *p*-values with and without a Bonferroni multiple comparisons correction are reported.

*Background about regularized linear regression using the Elastic Net framework.* Most LFP studies limit network hypotheses to one or two brain regions, use previously published LFP naming conventions to justify choosing the boundaries of frequency bands of interest *a priori*, and average activity across an entire oscillatory band to statistically test the relationship between LFPs and behavior. A major problem with this approach, however, is that it is not known how consistent the characteristics or the functions of oscillatory bands are across tasks or brain regions. Therefore, we chose to use a data-driven strategy to identify oscillation bands related to IA, given the absence of prior neural recordings collected during the IA test.

Applying data driven strategies to multi-site LFP data is challenging for two main reasons. First, if IA is regressed on all LFP frequency bands from all recording electrodes, the number of neural predictors (*p*) is much larger than the number of behavioral data points (*n*). This situation is called the *high-dimensionality* problem, or “p$\gg$n” problem. Second, neural oscillations occur in bands, so LFP activities in single 1-Hz frequency bands are highly correlated with LFP activity in other 1-Hz frequency bands. We will call this situation the *high-correlation* problem. Behaviorally-relevant oscillation bands cannot be validly identified by simply regressing IA on all power and coherence LFP parameters using the methods previously used in LFP studies because the *high-dimensionality* problem would lead to severe over-fitting and the *high-correlation* problem would lead to unstable results.

Regularized regression methods solve the *high-dimensionality* and *high-correlation* problems by penalizing regression coefficients so that extreme values indicative of over-fitting are removed, and by identifying the optimal model with good performance in predicting a response. There are three popular regularized regression frameworks named after their respective penalties: Lasso, Ridge, and Elastic-Net (ENET). Lasso regression would not be appropriate for LFP data because when regression coefficients are penalized using Lasso, only a small fraction (or one) of a set of highly correlated predictors are selected. Thus, rather than selecting an entire frequency band relevant to behavior, lasso regression would likely choose only 1 frequency unit out of a frequency band or set of frequency bands. That said, one advantage of Lasso regression is that it would select only a small number of neural predictors (or impose “sparsity”), which would highlight the strongest results. Ridge regression, on the other hand, could account for the correlation among neural predictors in LFP data, but it would not impose enough sparsity; it would assign a non-zero coefficient to every predictor in the model, making its results challenging to interpret. Elastic Net regression (ENET) is best suited for LFP data because it combines the desirable features of Lasso and Ridge regression by incorporating both types of penalties. Specifically, it selects a small fraction of the most informative neural predictors (preventing overfitting) while maintaining correlated neural predictors as a group (preventing over-sparsity). With ENET, unlike other published methods applied to LFP data, sparse representations of behaviorally-relevant sets of highly-correlated parameters can be identified in a reliable and stable fashion ([Zou and Hastie, 2005](#_ENREF_94)).

*Assessment of ENET model consistency*. To assess the consistency of our ENET procedure, we repeated the ENET-*Dark* analyses described in the main text with single animals removed from a model. We assessed the consistency of the predictors retained in these models compared to the full ENET model in three ways. First, to examine the consistency across models visually, we counted the number of times a parameter had a non-zero coefficient in the ENET results of each of the seven *Leave-one-rat-out* analyses and plotted the counts on the same axes as we examined the ENET-*Dark* results. If the coefficient was positive, it would be counted as “+1”. If the coefficient was negative, it would be counted as “-1”. Thus, a count of 7 for a predictor in these *Leave-one-rat-out analyses* would indicate that the ENET solutions of all seven models with the data from a single rat removed had a positive coefficient for that predictor. A count of -7 for a predictor in these *Leave-one-rat-out analyses* would indicate that the ENET solutions of all seven models with the data from a single rat removed had a negative coefficient for that predictor. A count of 5 for a predictor in these *Leave-one-rat-out analyses* could indicate that the ENET solutions of five models had a positive coefficient for that predictor while one model had a coefficient of 0, or it could indicate that the ENET solutions of six models had a positive coefficient for that predictor while one model had a negative coefficient.

Second, we calculated the percent of matches, and the true positive (TP), true negative (TN), false positive (FP), and false negative (FN) rates across of the ENET-*Leave-one-rat-out* analyses. “True positives” (TP) are predictors that were retained in both the ENET-*Dark* and the ENET-*Leave-one-rat-out* model under examination. “True negatives” (TN) are predictors whose coefficients were set to zero in both models. “False positives” (FP) are predictors with zero coefficients in the ENET-*Dark* analysis but non-zero coefficients in the ENET-*Leave-one-rat-out* model under examination. “False negatives” (FN) are predictors with non-zero coefficients in the ENET-*Dark* analysis, but zero coefficients in the ENET-*Leave-one-rat-out* model under examination. Let $\beta^{*}$ be the 5500×1 vector of the median regression coefﬁcients from the ENET-*Dark* analysis (the median of all 10 imputations). We define two sets of LFPs:

$T^{*}= \{j :\beta_{j}^{*}\neq0\}, N^{*}=\{j : \beta_{j}^{*}=0\}=T^{*c}, T^{*}\cup N^{*}=\{1,...,5500\}$ (2)

$T^{*}$represents the set of LFP parameters from the ENET-*Dark* analysis that have non-zero coefficients. $N^{*}$represents the set of LFPs parameters from the ENET-*Dark* analysis whose coefficients had a magnitude of zero. Let $\beta^{i}$ represent the set of LFPs parameters from the ENET-*Leave-one-rat-out* analysis for rat *i* (*i*= 1,…7). To compare $\beta^{i}$ to $\beta^{*}$, the percent matches are calculated as follows, where *n* = 7 and |*A*| represents the number of elements in set *A*:

${\%\mathrm{Matches}}_{TP}=\frac{\sum_{i}^{n} \left| T^{*}\cap T^{i} \right|}{\left| T^{*} \right|n}$, ${\%\mathrm{Matches}}_{TN}=\frac{\sum_{i}^{n} \left| N^{*}\cap N^{i} \right|}{\left| N^{*} \right|n}$,

${\%\mathrm{Matches}}_{FP}=\frac{\sum_{i}^{n} |N^{*}\cap T^{i}|}{|N^{*}|n}$, ${\%\mathrm{Matches}}_{FN}=\frac{\sum_{i}^{n} |T^{*}\cap N^{i}|}{|T^{*}|n}$

TP, TN, FP, and FN are defined in replication *i* of the ENET-*Leave-one-rat-out* cross-validation as:

$TP=\frac{|T^{*}\cap T^{i}|}{|T^{*}|}$, $TN=\frac{|N^{*}\cap N^{i}|}{|N^{*}|}$, $FP=\frac{|N^{*}\cap T^{i}|}{|N^{*}|}$, $FN=\frac{|T^{*}\cap N^{i}|}{|T^{*}|}$

$T^{i}$and $N^{i}$ are deﬁned by replacing $\beta^{*}$ by $\beta^{i}$ in (2) ([Hastie et al., 2009](#_ENREF_34)). The median (median absolute deviation) and mean (standard deviation) of these rates are then calculated across all seven cross-validation sets of the ENET-*Leave-one-rat-out* analysis.

Third, we assessed the consistency of the coefficient magnitudes across models by computing a root mean squared error (RMSE) for LFP parameters in the TP, FP, and FN categories of predictors. Let $T^{*}$ ∩ *T*^est^, $N^{*}$ ∩ *T*^est^, and $T^{*}$∗ ∩ *N*^est^ be the sets of LFP parameters representing the TP, FP, and FN, respectively, where “est” is *i* in replication *i* of leave-one-rat-out cross-validation analysis. RMSE for replication *i* is calculated by:

${RMSE}_{i,S}= \sqrt{\frac{1}{|S|}\sum_{j\in S} (\beta_{j}^{i}-}\beta_{j}^{i*})^{2}$,

**where *S* =** $T^{*}$ ∩ *T*^est^**,** $N^{*}$ ∩ *T*^est^**, and** $T^{*}$∗ ∩ *N*^est^**. This measure is interpreted on the scale of** $\beta^{*}$**, which has a maximum absolute value of approximately 10.**

**We also used the *leave-one-rat-out* analyses to estimate the predictive performance of our ENET-procedure. For each leave-one-rat-out cross validation set, the ENET procedure was run on 60 training data points. The median betas retrieved from the ten imputations of each procedure was then used to predict the IA of the rat who was left out of the training data set as follows:**

$$y_{\text{pred}}=\beta_{0}+X_{\text{test}}\beta,$$

**where** $y_{\text{pred}}$ **are the predicted IA values,** $X_{\text{test}}$ **is the *X* matrix for the rat who was left out, and** $\beta_{0}$ **and β are the median** $\beta_{\text{enet}}$ **values in (1) from all ten imputations of each ENET procedure. The predicted values were concatenated into one 70-item vector and compared to a concatenated vector of the true IA values. We estimated the predictive performance of our models using median absolute error (MAE) as follows:**

$$E_{i}=\text{median}\left( \left| y_{\text{test, }i}{-y}_{\text{pred, }i} \right| \right), T_{i}=\text{median}\left\{ \left| y_{\text{test, }i}{-\text{median}(y}_{\text{test, }i}) \right| \right\}, i= 1, \ldots, 7,$$

**where** $E_{i}$ **is the median absolute error in predicting IA values for rat *i* using the ENET model, and** $T_{i}$**is the median absolute error if the median value of the testing data was used as the predicted IA values for rat *i* (referred to as the null model). The MAE fraction, or fraction of error reduced by using the ENET model compared to the null model when predicting the concatenated IA values for all seven rats, is calculated by** ${E_{i}}/{T_{i}}$**. The lower the value of** ${E_{i}}/{T_{i}}$**, the better the comparative performance of the ENET model in predicting IA values.**

**Supplementary Figures and Tables.**

**SI Figure 1.** **Effects of** **individual rats on LFP parameters retained in the ENET-*Dark* model. A.** The results of the ENET-*Dark* analysis with all rats included (same as Fig. 6A of main text). **B.** To assess whether the results of the ENET–*Dark* analysis were driven by a single animal, we re-ran the ENET–*Dark* analysis seven times with the data from one rat left out of the analysis each time, and counted the number of times an LFP parameter was retained in the ENET solution of each *Leave-one-rat-out* cross-validation data set. The number of models that retained coefficients of the same valence for each individual parameter chosen are depicted on a scale from -7 to 7 (if all seven models had a positive coefficient in the leave-one-rat-out-analysis for a particular parameter, that parameter would be depicted by dark red; if all seven models had a negative coefficient in the leave-one-rat-out-analysis for a particular parameter, that parameter would be depicted by dark blue; if none of the models had a non-zero coefficient for a particular parameter, that parameter would be depicted in white).


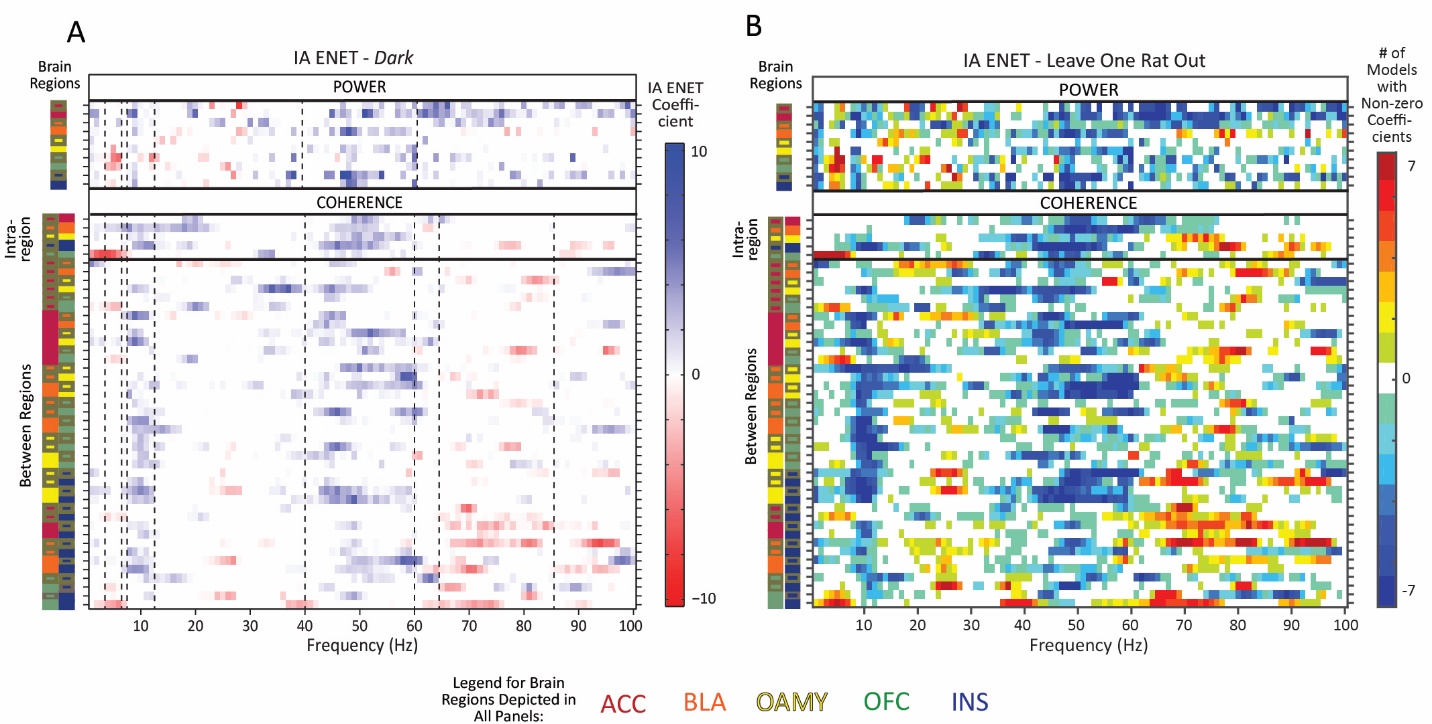


**SI Table 1:** The median (median absolute deviation, or “MAD”) and mean (standard deviation, or “SD”) true positive rate of predictors that had coefficients with absolute values greater than 6, 5, 4, 3, 2, or 1 in the ENET-*Dark* analysis.

| When Coefficients in ENET-*Dark* were greater than… | % with non-zero coefficients in cross-validation sets | True positive rate across cross-validation sets (median, MAD) | True positive rate across cross-validation sets (mean, SD) |
| --- | --- | --- | --- |
| 6 | 97.71 | 1.00 (0.04) | 0.98 (0.06) |
| 5 | 96.49 | 1.00 (0.06) | 0.96 (0.09) |
| 4 | 94.31 | 0.99 (0.07) | 0.94 (0.12) |
| 3 | 89.53 | 0.97 (0.12) | 0.90 (0.17) |
| 2 | 84.73 | 0.94 (0.15) | 0.85 (0.20) |
| 1 | 77.57 | 0.87 (0.17) | 0.78 (0.22) |

**SI Table 2:** Medians (median absolute deviation) and means (standard deviation) of the absolute values of the magnitudes of False Negative and False Positive coefficients in the *Leave-one-rat-out* analysis.

|  | False Negatives | False Positives |
| --- | --- | --- |
| Median Magnitude of the Coefficients (MAD) | 0.52 (0.85) | 0.44 (0.72) |
| Mean Magnitude of the Coefficients (SD) | 0.88 (0.96) | 0.74 (0.90) |

**­­**

**SI Figure 2.** **Evaluation of** **ENET Model fit.** **A.** Scatter plot of the true measured values of IA (defined as change in dark preference) versus the IA values predicted by the ENET- *Dark* model fit (no testing data withheld). Strongly positive or negative changes in preference for the dark chamber are underestimated. **B.** Scatter plot of the true measured values of IA versus the predicted values of IA in the training set of each leave-one-rat out model. **C.** Plot of the true measured values of IA versus the predicted values of IA in the testing set of each leave-one-rat out model.


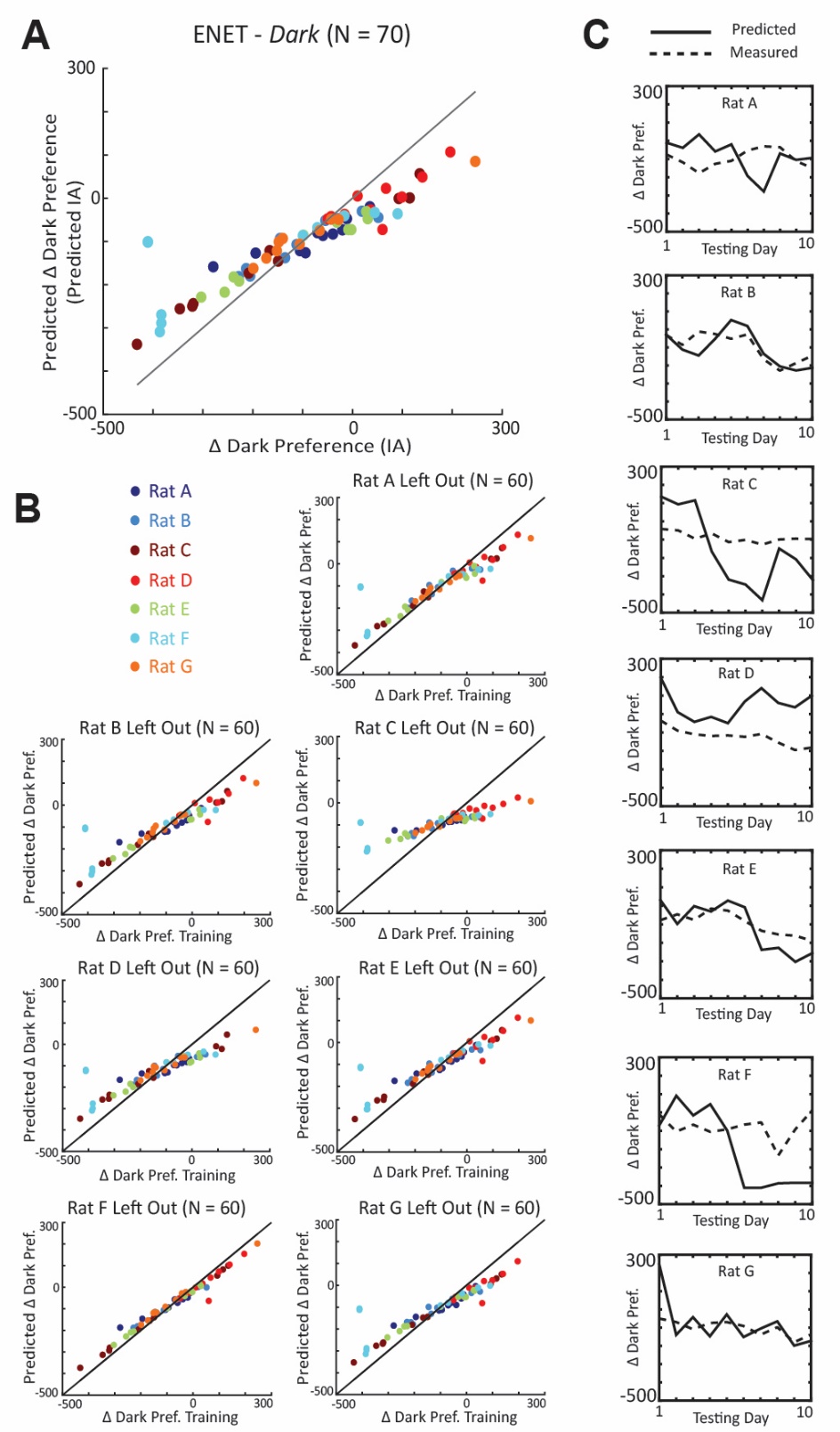


­­­**Relationships between Grooming, Social Investigation, and LFPs**

Since there were significant negative Pearson correlations between IA (Δ dark chamber preference) and changes in percent dark chamber grooming (“Δ % Dark Grooming”, *r* = -0.37, *p* < .01) and percent dark chamber social investigation (“Δ % SI”, *r* = -0.32, *p* < .01), we examined the relationships between LFP parameters and Δ % Dark Grooming and Δ % SI to determine whether such relationships were likely to explain the ENET-*Dark* results. We examined this by plotting the Spearman correlation coefficients between all 5500 LFP predictors and individual differences in Δ % Dark Grooming and Δ % Dark SI, and by running ENET analysis that replaced the dependent variable IA with either Δ % Dark Grooming (ENET – *Dark Grooming*) or Δ % Dark SI (ENET – *Dark SI*). Positive relationships between Δ % Dark Grooming or Δ % Dark SI and LFP parameters (indicated in purple in SI Fig. 3 and 4) might explain positive relationships between IA and LFP parameters (indicated in blue in the IA ENET-*Dark* plots of the main text). Negative relationships between Δ % Dark Grooming or Δ % Dark SI and LFP parameters (indicated in orange in SI Fig. 3 and 4) might explain negative relationships between IA and LFP parameters (indicated in red in the IA ENET-*Dark* plots of the main text).

SI Fig. 3A and 3B suggest that Δ % Dark Grooming might account for some scattered positive relationships between low gamma coherence and IA, and some negative relationships between high gamma coherence and IA.

SI Fig. 4A and 4B suggest that Δ % Dark SI might account for some of the positive relationships between alpha power or coherence and IA, and some of the positive relationships between low and high gamma power and IA. Other less dramatic confounds might exist in relationships between low and high gamma coherence and IA as well.

Note that the ENET procedure uses ***y*** vectors that are centered, but not standardized. Thus, although the presence or absence of LFP predictors can be compared across the IA ENET-*Dark,* ENET-*Dark Grooming,* ENET- *Dark SI*, and IA-Residuals ENET-*Dark* models, the magnitude of the coefficients should not be compared directly. The limits of the color bars for each plot are chosen according the maximum absolute value of the coefficients being depicted.

**SI Figure 3.** **Relationships between grooming and LFPs.** **A.** Spearman correlation coefficients between all 5500 LFP predictors and individual differences in Δ % Dark Grooming. **B.** ENET – *Dark Grooming.* Δ % Dark Grooming was regressed on LFP activity, and the ENET framework was applied to regularize (or penalize) the regression coefficients. The maximum and minimum values of the color bar were determined by the maximum and minimum values of the ENET coefficients. For **A.** and **B.**, brain regions pairs are indicated by color-coded squares, and no pairs are repeated. Left hemisphere regions are outlined in gray, right hemisphere regions have no outline.


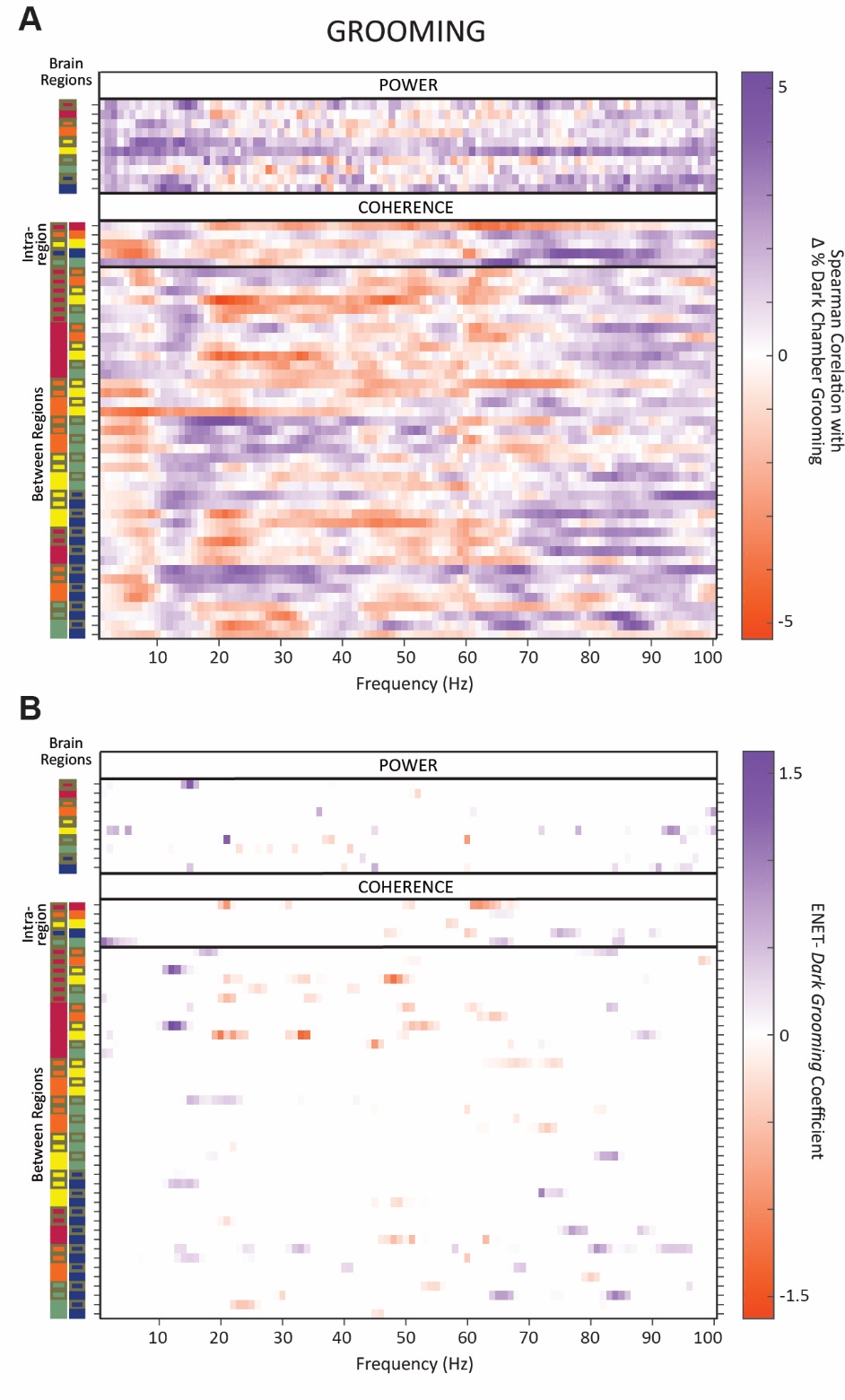


**SI Figure 4.** **Relationships between social investigation and LFPs.** **A.** Spearman correlation coefficients between all 5500 LFP predictors and individual differences in Δ % Dark Social Investigation (SI). **B.** ENET – *Dark SI.* Δ % Dark SI was regressed on LFP activity, and the ENET framework was applied to regularize (or penalize) the regression coefficients. The maximum and minimum values of the color bar were determined by the maximum and minimum values of the ENET coefficients. For **A.** and **B.**, brain regions pairs are indicated by color-coded squares, and no pairs are repeated. Left hemisphere regions are outlined in gray, right hemisphere regions have no outline.


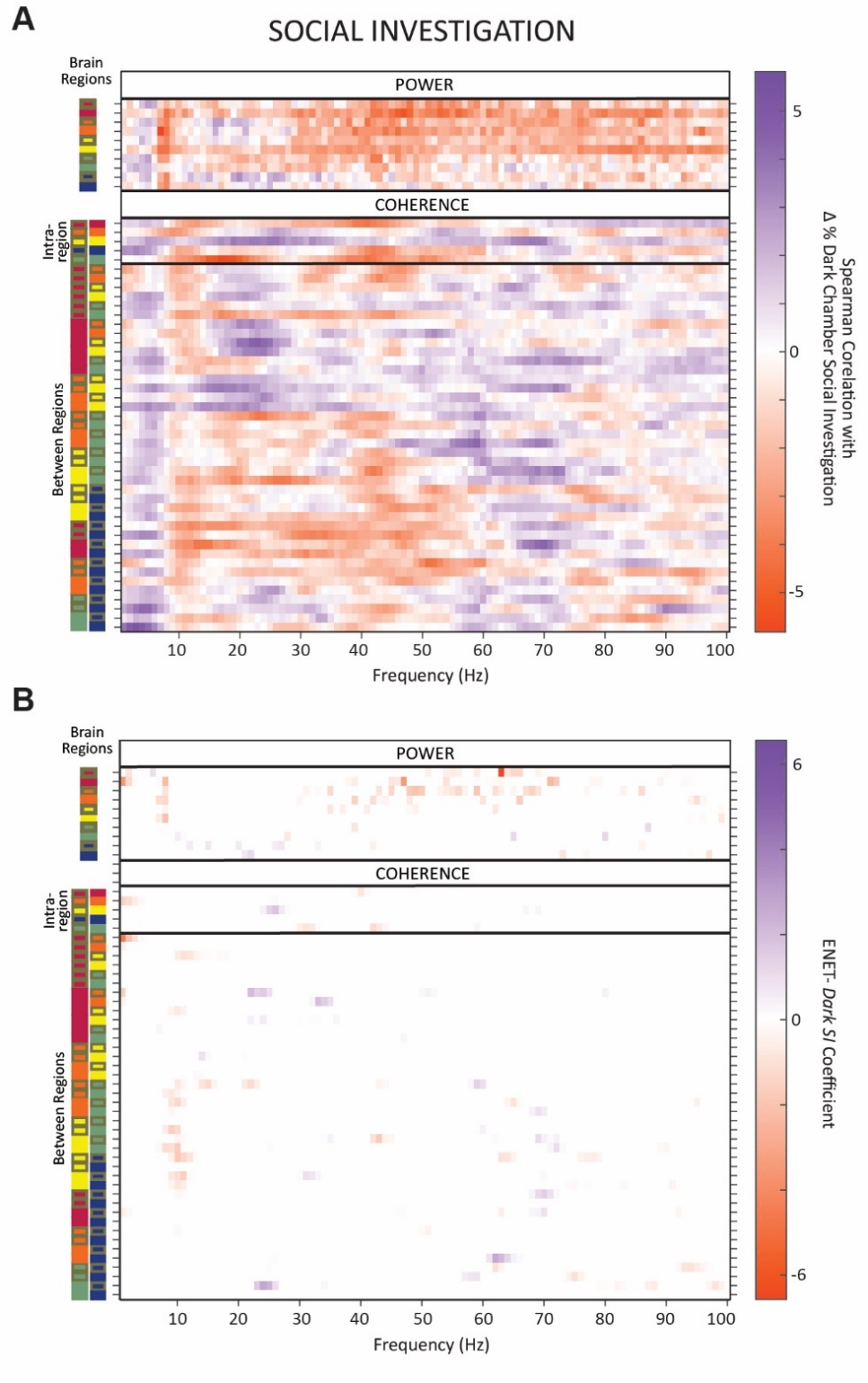


**SI Figure 5.** **IA-Residuals analysis.** **A.** IA ENET – *Dark.* **B.** IA-Residuals ENET – *Dark.* IA was regressed on Δ % Grooming and Δ % SI, and the residuals (“IA-residuals”) were saved. The ENET-Dark Analysis was repeated using these residuals as the dependent variable to determine if there were still relationships between IA and LFP parameters, once the shared variance between IA, Grooming, and SI was removed. All parameters were modeled jointly, brain regions pairs are indicated by color-coded squares, and no pairs are repeated. Left hemisphere regions are outlined in gray, right hemisphere regions have no outline. The minimum and maximum values of the color bar were set to the minimum and maximum values of the coefficients in each ENET model to make it easier to visually compare which predictors were retained in each model, but note that the magnitude of the IA-Residuals ENET – *Dark* analysis are about half the size of the IA ENET – *Dark* coefficients.


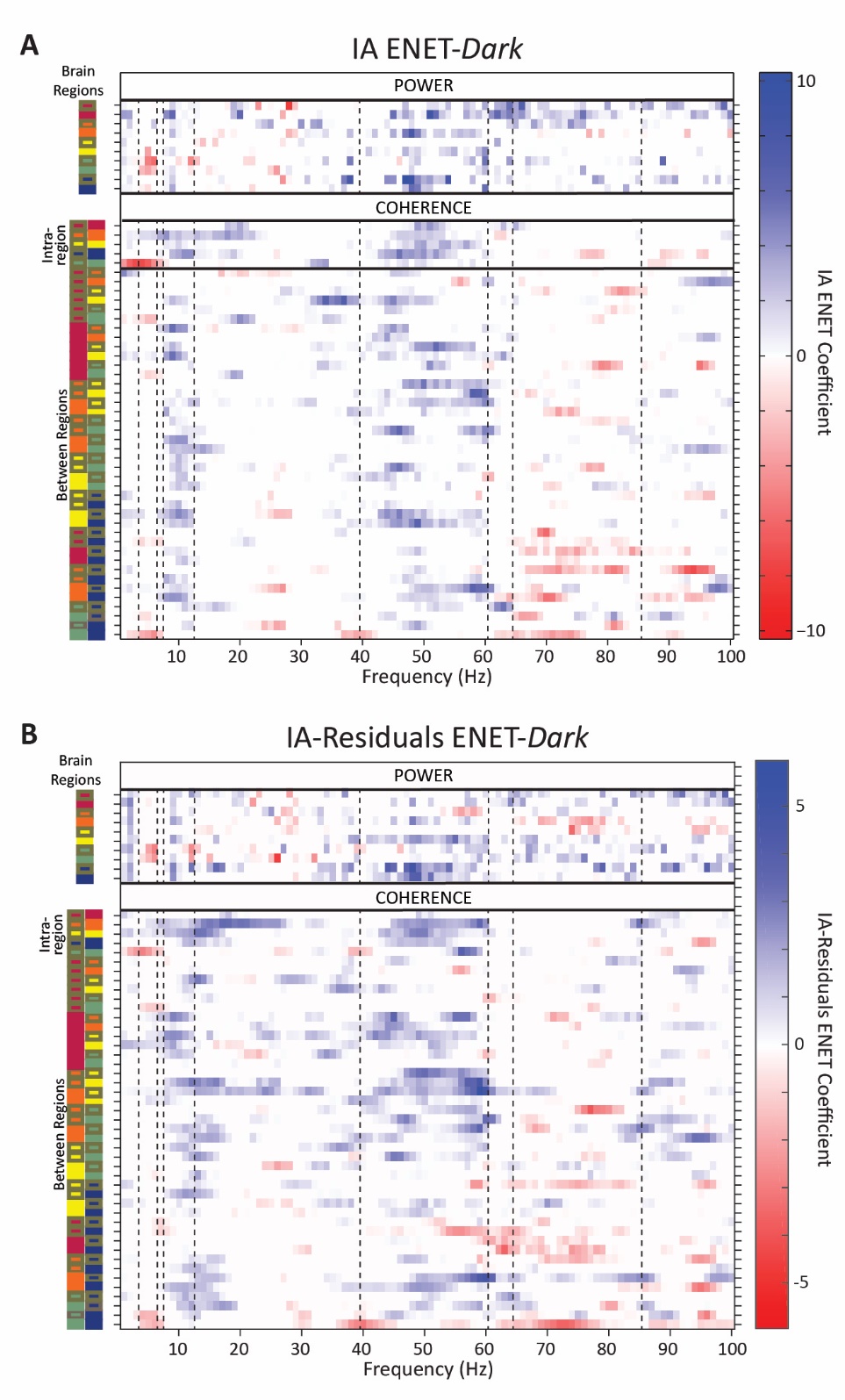


**SI Figure 6.** **IA-Residuals ENET-*Dark* *MRL*.** The predictors in this ENET analysis included the same power predictors as the IA-Residuals ENET-*Dark* analysis, but the coherence predictors were replaced with MRL measurements on each testing day compared to the baseline average. All parameters were modeled jointly. Brain regions pairs are indicated by the color-coded squares; no pairs are repeated. Left hemisphere regions are outlined in gray, right hemisphere regions have no outline.


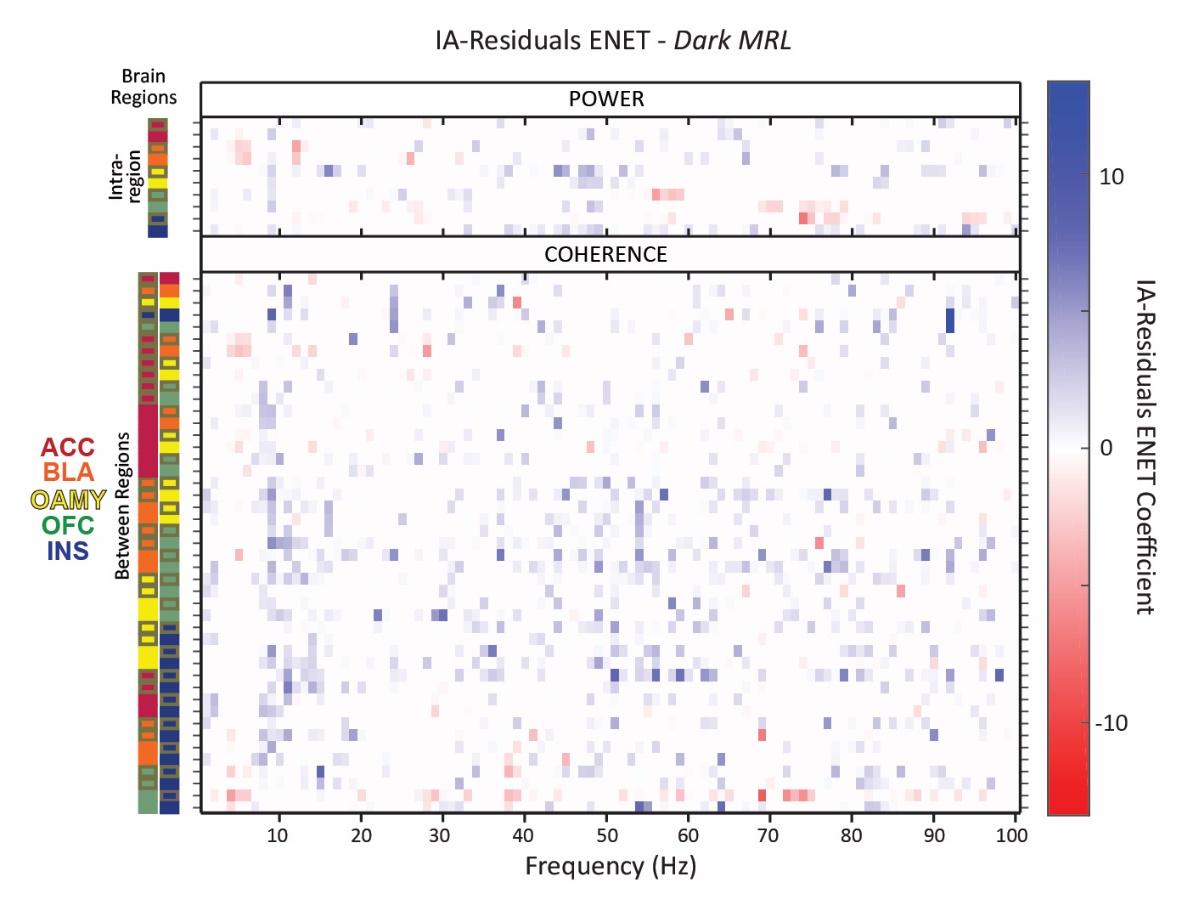

Supplement: Supplementary file 1 [file BRB3-7-e00710-s001.docx]
